# Magnetic and luminescent coordination networks based on imidazolium salts and lanthanides for sensitive ratiometric thermometry

**DOI:** 10.3762/bjnano.9.259

**Published:** 2018-10-30

**Authors:** Pierre Farger, Cédric Leuvrey, Mathieu Gallart, Pierre Gilliot, Guillaume Rogez, João Rocha, Duarte Ananias, Pierre Rabu, Emilie Delahaye

**Affiliations:** 1 Institut de Physique et Chimie des Matériaux de Strasbourg, Université de Strasbourg, CNRS UMR 7504, F-67034 Strasbourg Cedex 2, France; 2 Department of Chemistry, CICECO, University of Aveiro, 3810-193 Aveiro, Portugal

**Keywords:** coordination network, imidazolium salt, lanthanides, magnetism, thermometry

## Abstract

The synthesis and characterization of six new lanthanide networks [Ln(L)(ox)(H_2_O)] with Ln = Eu^3+^, Gd^3+^, Tb^3+^, Dy^3+^, Ho^3+^ and Yb^3+^ is reported. They were synthesized by solvo-ionothermal reaction of lanthanide nitrate Ln(NO_3_)_3_·*x*H_2_O with the 1,3-bis(carboxymethyl)imidazolium [HL] ligand and oxalic acid (H_2_ox) in a water/ethanol solution. The crystal structure of these compounds has been solved on single crystals and the magnetic and luminescent properties have been investigated relying on intrinsic properties of the lanthanide ions. The synthetic strategy has been extended to mixed lanthanide networks leading to four isostructural networks of formula [Tb_1−_*_x_*Eu*_x_*(L)(ox)(H_2_O)] with *x* = 0.01, 0.03, 0.05 and 0.10. These materials were assessed as luminescent ratiometric thermometers based on the emission intensities of ligand, Tb^3+^ and Eu^3+^. The best sensitivities were obtained using the ratio between the emission intensities of Eu^3+^ (^5^D_0_→^7^F_2_ transition) and of the ligand as the thermometric parameter. [Tb_0.97_Eu_0.03_(L)(ox)(H_2_O)] was found to be one of the best thermometers among lanthanide-bearing coordination polymers and metal-organic frameworks, operative in the physiological range with a maximum sensitivity of 1.38%·K^−1^ at 340 K.

## Introduction

Metal-organic coordination networks have been the subject of considerable research in the last years as evidenced by the increasing number of papers published in the field [1]. Indeed, the possibility of combining different properties by judicious choice of the organic and inorganic moieties makes these systems good candidates for the elaboration of (multi)functional architectures [2–3]. Among the various functionalities that can be envisioned for this class of hybrid compounds, the elaboration of luminescent networks is interesting in term of potential applications in lighting, display, sensing, biomedicine and for optical devices [4–12].

Luminescent coordination networks can be obtained either by the use of specific luminescent organic ligands or by the use of main-group elements, d^10^ transition metals or of trivalent lanthanide ions for the inorganic moiety [13–14]. The luminescent properties of the trivalent lanthanide ions are particularly interesting since they cover a large range of emission from the ultraviolet (Gd^3+^) to near-infrared (Pr^3+^, Nd^3+^, Ho^3+^, Er^3+^, Yb^3+^) through the visible domain (Pr^3+^, Sm^3+^, Eu^3+^, Tb^3+^, Dy^3+^, Tm^3+^). It confers to lanthanide-based networks a large tunability of emission properties, which is very useful for the elaboration of light-emitting devices or for biomedical applications [15]. Moreover, due to the narrowness and the hypersensitivity of their transitions, lanthanide-based networks can also find utility for the sensing of gases, vapors or small molecules [9,16]. In the case of mixed lanthanide coordination networks, the luminescent properties can be used to synthesize temperature probes with possible applications in the aerospace area, safety and health [17–18].

Beside luminescent properties, lanthanide ions exhibit large magnetic moment and strong magnetic anisotropy, which might have potential applications of lanthanide-based networks in information storage, quantum computing and spintronics [19–23].

Most of these lanthanide-based networks are obtained with neutral organic ligands such as benzene-1,4-dicarboxylate (1,4-bdc) [24], benzene-1,3,5-tricarboxylate (TMA) [25], pyridine-2,5-dicarboxylate (2,5-H_2_pdc) [24] or 1*H*-2-propyl-4,5-imidazoledicarboxylate (pimda) [26]. Only few examples of lanthanide-based networks obtained with charged ligands are reported in the literature [27–30]. Following this last point, we have chosen to synthesize lanthanide-based networks from positively charged imidazolium dicarboxylate salts [31–34]. Contrarily to the classical imidazolium salts or ionic liquids used in ionothermal syntheses [35–40], the functionalization of imidazolium moieties with coordinating functions reduces the influence of the imidazolium salt on the ligand for a better rationalization of the synthesis [31–34].

We report in this paper the synthesis and the characterization of six new networks obtained from an imidazolium dicarboxylate salt, oxalic acid and lanthanide ions. The structure of these networks has been solved by single crystal X-ray diffraction and their physical properties (magnetism and luminescence) have been investigated. We establish that these networks show antiferromagnetic interactions. The study of the luminescent properties evidences the presence of well-defined transitions characteristic for the considered lanthanide. These results have prompted us to extend our strategy to the synthesis of mixed lanthanide networks with four different ratios Tb^3+^/Eu^3+^. The powder X-ray diffraction analysis indicates that these mixed lanthanide networks are isostructural to the parent homolanthanide compounds. The temperature-resolved photo-luminescent properties of the latter indicate possible applications in thermometry.

## Results and Discussion

### Synthesis

The 1,3-bis(carboxymethyl-)-imidazolium ligand [HL] was synthesized according to protocols published in the literature [41–42].

Single crystals and homogeneous powders of [Ln(L)(ox)(H_2_O)] were obtained with Ln = Eu^3+^, Gd^3+^, Tb^3+^, Dy^3+^, Ho^3+^ and Yb^3+^ by reacting a water/ethanol solution of the lanthanide nitrate and oxalic acid (H_2_ox) with [HL]. The mixture was sealed in a Teflon-lined stainless steel autoclave and heated at 393 K for 72 h. After cooling to room temperature, the autoclaves were opened and crystals were filtered and washed with ethanol. The yields of the reactions range from 36 to 59 %. Similar reactions were carried out with Nd^3+^ and Sm^3+^ ions leading to different structures [31]. In addition, in the case of Nd^3+^ and Sm^3+^, various crystalline compounds were obtained depending on whether oxalic acid was added or not. When oxalic acid was not added in situ formation of the oxalate ligand has been observed. The peculiar behavior of these two ions compared to others can be explained by their place in the first part of the lanthanide series [43]. In the case of Eu^3+^, Gd^3+^, Tb^3+^, Dy^3+^, Ho^3+^ and Yb^3+^, described in the present work, the direct reaction between lanthanide nitrate and [HL], without addition of oxalic acid, did not give crystalline compounds.

### Characterization of the homolanthanide [Ln(L)(ox)(H_2_O)] compounds with Ln = Eu^3+^, Gd^3+^, Tb^3+^, Dy^3+^, Ho^3+^ and Yb^3+^

Single crystal X-ray analysis of the [Ln(L)(ox)(H_2_O)] compounds with Ln = Eu^3+^, Gd^3+^, Tb^3+^, Dy^3+^, Ho^3+^ and Yb^3+^ reveal that the six compounds are isostructural. All compounds are obtained as colorless crystals and crystallize in the monoclinic space group *P*2_1_/*a* (no. 14). Crystal data for these series of compounds are collected in Table 1 and Table 2.

**Table 1:** Crystallographic data for [Ln(L)(ox)(H_2_O)] compounds with Ln = Eu^3+^, Gd^3+^ and Tb^3+^.^a^

	[Eu(L)(ox)(H_2_O)]	[Gd(L)(ox)(H_2_O)]	[Tb(L)(ox)(H_2_O)]

chemical formula	C_9_H_9_N_2_O_9_Eu	C_9_H_9_N_2_O_9_Gd	C_9_H_9_N_2_O_9_Tb
molar mass [g·mol^−1^]	441.14	446.43	448.10
crystal system	monoclinic	monoclinic	monoclinic
space group	*P*2_1_/*a*	*P*2_1_/*a*	*P*2_1_/*a*
*a* [Å]	9.212(3)	9.224(4)	9.246(3)
*b* [Å]	13.228(4)	13.226(4)	13.219(9)
*c* [Å]	10.9893(17)	10.950(2)	10.904(3)
α [°]	90	90	90
β [°]	111.491(18)	111.48(2)	111.63(2)
γ [°]	90	90	90
*Z*	4	4	4
*T* [K]	293(2)	293(2)	293(2)
μ (Mo Kα) [mm^−1^]	5.044	5.366	5.716
reflection collected	11538	6602	14582
independent reflections	2854	2825	2840
data/restraints/parameters	2854/3/196	2825/3/196	2840/3/196
R1, wR2 [*I* > 2σ(*I*)]	0.0358, 0.0690	0.0268, 0.0458	0.0427, 0.0769
R1, wR2 [all data]	0.0579, 0.0770	0.0430, 0.0501	0.0627, 0.0850
GOOF	1.063	1.092	1.094
largest diff. peak and hole (e·Å^−3^)	1.297, −1.365	0.681, −0.689	1.868, −1.838

^a^The relatively high values of the residual density can be explained by the difficulty to isolate single crystals. Indeed SEM images reveal the presence of relatively small and entangled crystals (see Figure S3, Supporting Information File 1).

**Table 2:** Crystallographic data for [Ln(L)(ox)(H_2_O)] compounds with Ln = Dy^3+^, Ho^3+^ and Yb^3+^.^a^

	[Dy(L)(ox)(H_2_O)]	[Ho(L)(ox)(H_2_O)]	[Yb(L)(ox)(H_2_O)]

chemical formula	C_9_H_9_N_2_O_9_Dy	C_9_H_9_N_2_O_9_Ho	C_9_H_9_N_2_O_9_Yb
molar mass [g·mol^−1^]	451.68	454.11	462.22
crystal system	monoclinic	monoclinic	monoclinic
space group	*P*2_1_/*a*	*P*2_1_/*a*	*P*2_1_/*a*
*a* [Å]	9.191(4)	9.228(10)	9.193(2)
*b* [Å]	13.188(4)	13.185(4)	13.097(3)
*c* [Å]	10.85(5)	10.862(8)	10.721(5)
α [°]	90	90	90
β [°]	111.63(3)	111.95(6)	112.19(3)
γ [°]	90	90	90
*Z*	4	4	4
*T* [K]	293(2)	293(2)	293(2)
μ (Mo Kα) [mm^−1^]	6.137	6.479	7.843
reflection collected	13140	13992	7321
independent reflections	2802	2814	2735
data/restraints/parameters	2802/3/196	2814/3/196	2735/3/196
R1, wR2 [*I* > 2σ(*I*)]	0.0336, 0.0573	0.0494, 0.0751	0.0516, 0.1119
R1, wR2 [all data]	0.0517, 0.0624	0.0854, 0.0840	0.0914, 0.1319
GOOF	1.122	1.133	1.031
largest diff. peak and hole (e·Å^−3^)	0.742, −1.382	1.164, −1.034	2.751, −2.662

^a^The relatively high values of the residual density can be explained by the difficulty to isolate single crystals. Indeed SEM images reveal the presence of relatively small and entangled crystals (see Figure S3, Supporting Information File 1).

**Figure 1:**
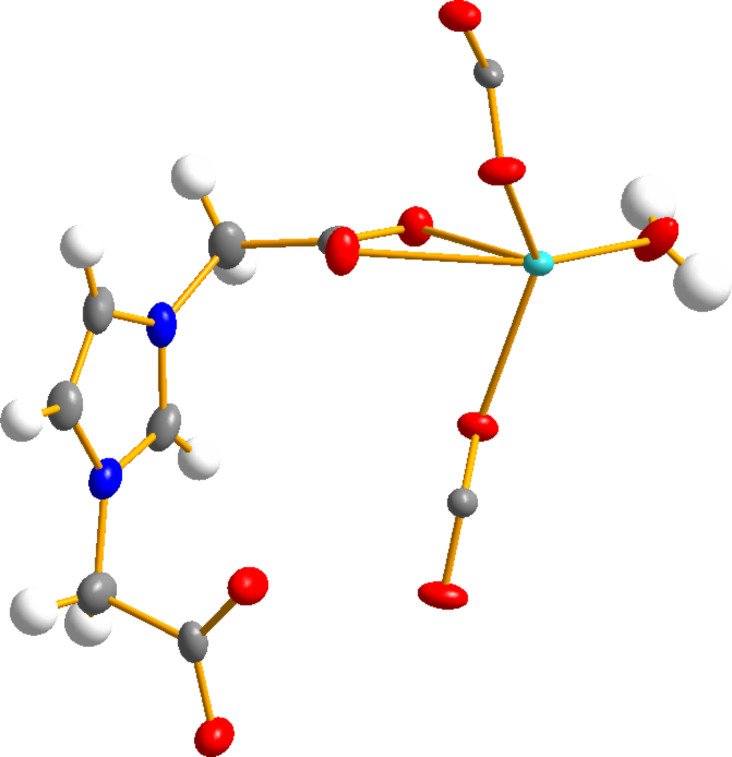
Ellipsoid view of the asymmetric unit of [Gd(L)(ox)(H_2_O)] (Gd in green, C in grey, O in red, N in blue and H in white).

The asymmetric unit contains one Ln^3+^ ion, one [L]^−^ ligand, two half-oxalate ligands and one coordinating water molecule (Figure 1). Ln^3+^ ions are surrounded by nine oxygens with four oxygen atoms coming from one and same carboxylate function of two different [L]^−^ ligands, one from the water molecule and four from two different oxalate ligands. The coordination environment of Ln^3+^ ions is a tricapped trigonal prism (Figure S1, Supporting Information File 1) with Ln–O distances similar to those observed in structurally related compounds [44–45]. These distances decrease progressively with the size of the lanthanide ion in agreement with the lanthanide contraction effect (Table S1, Supporting Information File 1). The same tendency is observed with the shortest Ln–Ln distances, which correspond to two Ln^3+^ ions connected by an oxalate ligand (Table S1, Supporting Information File 1).

**Figure 2:**
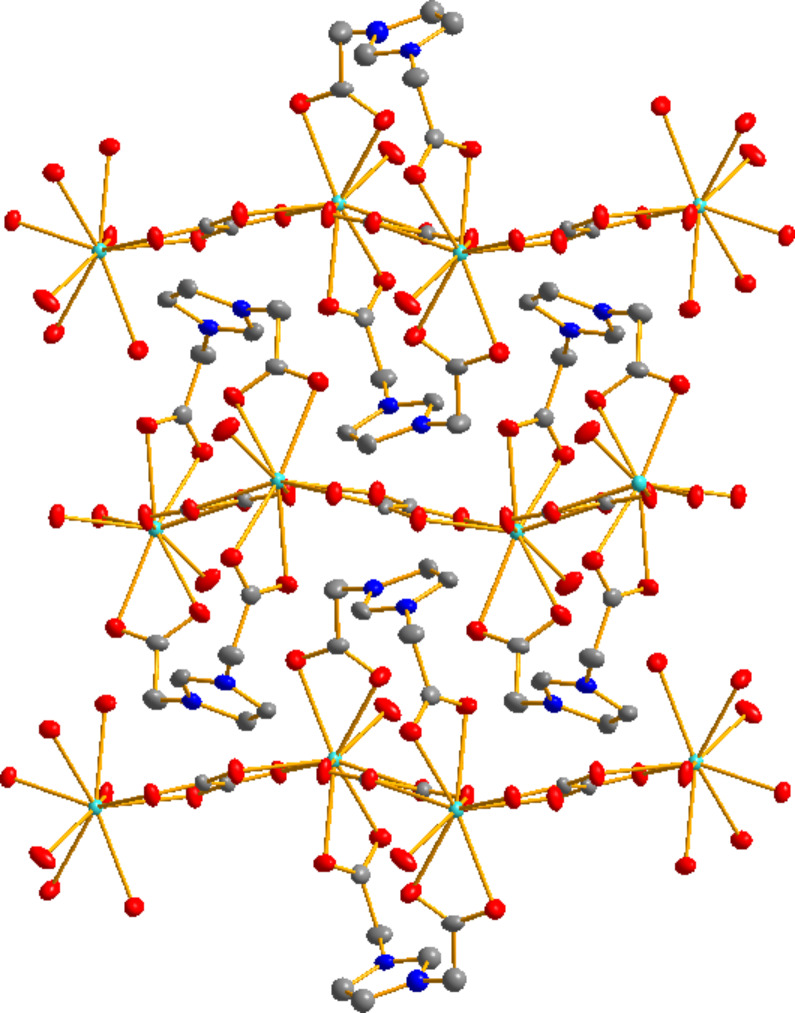
Selected packing view of the crystal structure of [Gd(L)(ox)(H_2_O)] along the c-axis (Gd in green, C in grey, O in red, N in blue). H atoms have been omitted for clarity.

Two separate Ln^3+^ ions are connected through an oxalate ligand in a bis-bidentate bridging coordination mode forming undulating chains along the *a*-axis (Figure 2). The Ln^3+^ ions are connected to the carboxylate functions of the [L]^−^ ligand in a bidentate chelate mode. The cohesion between these chains is realized through H bonding between H atoms of the coordinated water molecules and O atoms of the carboxylate functions.

Beside the single crystal analysis, the homogeneity of the six samples was checked by powder X-ray diffraction. As shown in Figure S2 (Supporting Information File 1), the experimental powder patterns fit well with the patterns calculated from the single crystal structure and show no additional phases.

In order to investigate the thermal stability, thermogravimetric analysis (TGA) was realized under air stream from 25 to 900 °C (Figure S4, Supporting Information File 1). The weight loss corresponding to the elimination of the coordinated water molecule occurs between 130 and 310 °C (step 1). The second weight loss between 310 and 750 °C (step 2) is associated to the combustion of the organic moieties (oxalate and [L]^−^ ligands), concomitant with the formation of oxide (Ln_2_O_3_ was identified by powder X-ray diffraction in the final product). The total weight loss is in good agreement with the calculated values (Table S2, Supporting Information File 1).

The infrared spectra of the six compounds are similar (Figure S5, Supporting Information File 1). The broad band around 3250 cm^−1^ and the one at 1672 cm^−1^ are ascribed to the coordinated water (stretching and bending vibration modes, respectively). The vibration bands of the aromatic and aliphatic C–H bonds are observed in the range 3150–3050 cm^−1^ and 3050–2950 cm^−1^, respectively. The characteristic frequencies of the coordinating carboxylate functions are observed at 1627 and 1571 cm^−1^ (antisymmetric vibration bands) and at 1411 and 1431 cm^−1^ (symmetric vibration bands). It leads to Δν (Δν = ν_antisym_ − ν_sym_) equal to 216 and 140 cm^−1^ in agreement with a bis-bidentate bridging coordination mode of the oxalate ligand and a bidentate chelate coordination mode of the carboxylate functions of the [L]^−^ ligand, respectively [46–47].

### Magnetic properties

**Figure 3:**
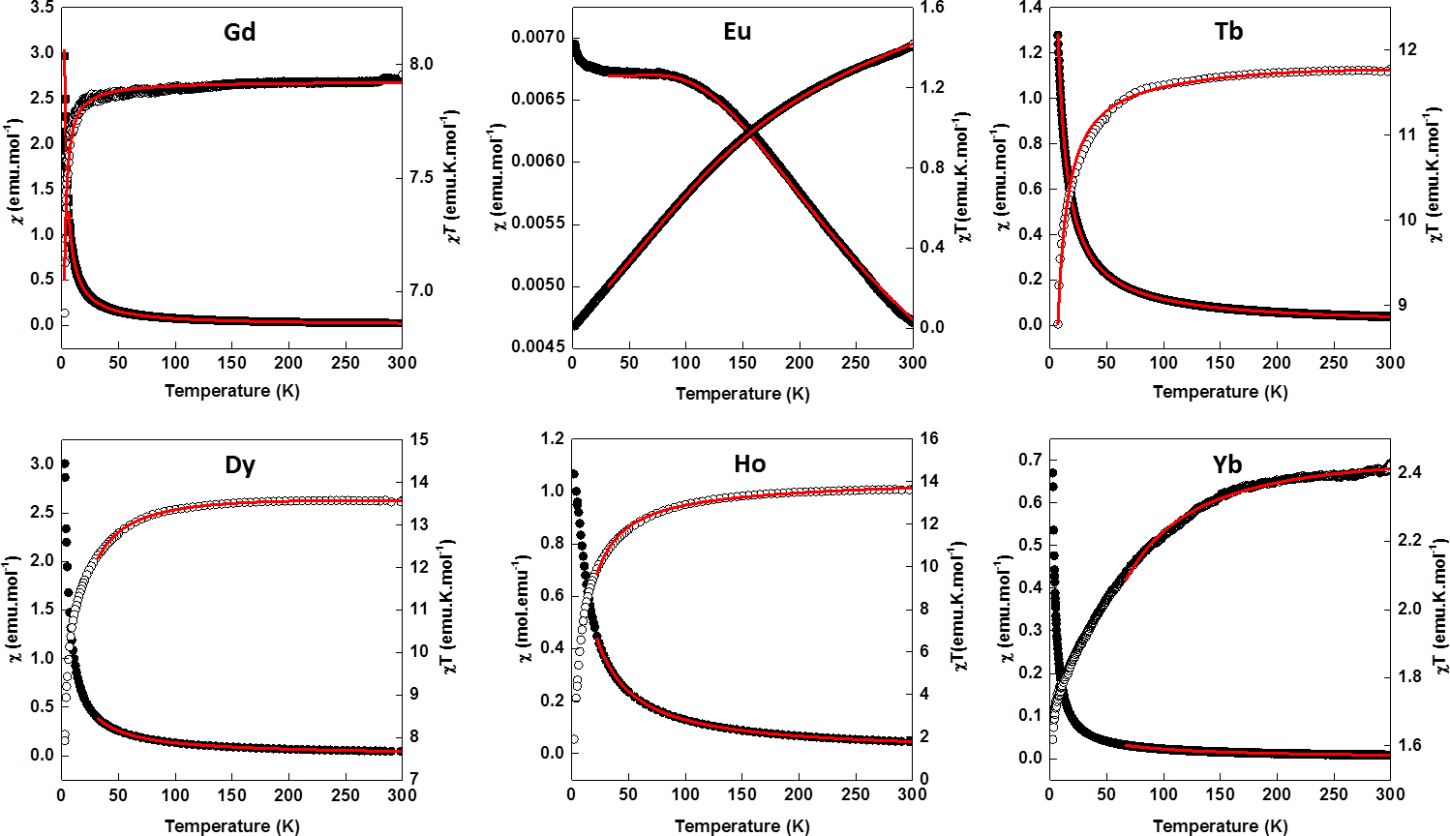
Plots of χ (closed circles) and χ*T* (open circles) as functions of *T* for [Ln(L)_2_(ox)(H_2_O)] with Ln = Gd^3+^, Eu^3+^, Tb^3+^, Dy^3+^, Ho^3+^ and Yb^3+^. The full lines correspond to the fit of the data using the expressions discussed in the text and given in Supporting Information File 1.

The magnetic behavior of the six compounds [Ln(L)(ox)(H_2_O)] have been studied in the temperature range of 1.8–300 K under a 0.5 T dc magnetic field. The magnetic susceptibilities and products χ*T* are presented as functions of the temperature in Figure 3.

At 300 K, the value of χ*T* for the compound [Gd(L)(ox)(H_2_O)] is 7.88 emu·K·mol^−1^, which agrees well with the theoretical value for spin-only *S* = 7/2 Gd^3+^ ions. The χ*T* product remains almost constant above 30 K and then decreases down to 6.7 emu·K·mol^−1^ at 1.8 K. This decrease suggests the occurrence of antiferromagnetic coupling between neighboring gadolinium centers. Since [Gd(L)(ox)(H_2_O)] is constituted of linear chains of Gd^3+^ ions with large spin moment, *S* = 7/2, we evaluated the magnetic coupling, *J*, between neighboring Gd^3+^ ions by using the Fisher expression for classical spin chains [48–49]:


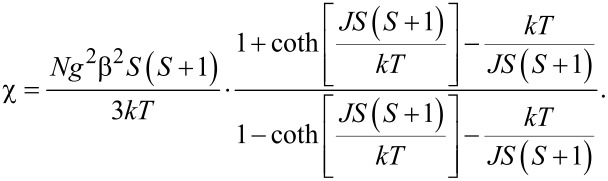


In the above expression, *N* is the Avogadro number, *g* is the Landé factor, β is the Bohr magneton, *k* is the Boltzmann constant, *S* is the spin moment, *J* is the magnetic coupling constant and *T* is the temperature. The simultaneous fitting of the susceptibility and the χ*T* product with the Fischer expression above lead to refined values of *g* = 2.00(1) and *J* = −0.026 cm^−1^. The *g* value was left free during fitting and is in line with the tabulated *g* values for Gd ions [50]. The absolute value and the sign of *J* support the presence of weak antiferromagnetic interactions in [Gd(L)(ox)(H_2_O)], in agreement with results reported in previous works [51–53]. The magnetic exchange coupling between lanthanide ions is usually weak, due to limited extension of the 4f orbitals.

For [Eu(L)(ox)(H_2_O)], the χ*T* product decreases continuously from 1.4 emu·K·mol^−1^ at 300 K to 0 emu·K·mol^−1^ at 1.8 K. This behavior is typical for Eu^3+^ ions for which the *^7^*F ground term is split in seven *^7^*F*_J_* (0 ≤ *J* ≤ 6) states because of spin–orbit coupling [54–55]. The spin–orbit coupling constant, λ, can be evaluated considering isotropic isolated Eu^3+^ ions parametrized with the appropriate expression (E1 in Supporting Information File 1) for the isotropic susceptibility of Eu^3+^ ions [54–55].

A very good fit of the experimental susceptibility and the χ*T* product of [Eu(L)(ox)(H_2_O)] was obtained above 25 K giving the refined value of λ = 309.00(4) cm^−1^. This value is consistent with the value determined from the luminescence measurements (see below) and confirms that considering only the isotropic component of the susceptibility is a good approximation to analyze the magnetic data [54].

The behavior of [Tb(L)(ox)(H_2_O)] is also typical for quasi-isolated Tb^3+^ ions with χ*T* = 11.75 emu·K·mol^−1^ at 300 K (expected value: 11.82 emu·K·mol^−1^ with *g* = 1.5) [48,50]. When decreasing the temperature, the χ*T* product remains constant until 100 K and then decreases to 4.50 emu·K·mol^−1^ at 1.8 K. This decay is due to the depopulation of the low-lying *J* states arising from the splitting of the *^7^*F ground term under spin–orbit coupling. In order to determine the spin–orbit coupling, λ, it was necessary to take into account an antiferromagnetic interaction between neighboring Tb^3+^ ions using a mean-field approach, in addition to the intrinsic behavior of isolated Tb^3+^ ions (E5 in Supporting Information File 1) [56].

Subsequently, a good fit of the magnetic data was obtained on the whole temperature range with λ = −303(75) cm^−1^ and *zJ*′ = −0.106(1) cm^−1^. The obtained λ value is consistent with other values reported in the literature for compounds containing isolated Tb^3+^ ions [57–58].

In the case of [Dy(L)(ox)(H_2_O)], [Ho(L)(ox)(H_2_O)] and [Yb(L)(ox)(H_2_O)] the χ*T* values at 300 K are 13.82, 13.61 and 2.48 emu·K·mol^−1^, in line with the theoretical values for isolated Dy^3+^ ions (14.17 emu·K·mol^−1^ with *g* = 1.33) [50] Ho^3+^ ions (14.07 emu·K·mol^−1^ with *g* = 1.25) [50,59], and Yb^3+^ ions (theoretical value of 2.57 emu·K·mol^−1^ with *g* = 1.14) [48,50]. Upon cooling, the χ*T* product of [Dy(L)(ox)(H_2_O)] remains nearly constant until 170 K and then decreases to 9.90 emu·K·mol^−1^ at 1.8 K. For the Ho analogue [Ho(L)(ox)(H_2_O)], the χ*T* product decreases slowly between 300 and 100 K, and a steeper decrease is observed from 13.67 emu·K·mol^−1^ at 100 K to 2 emu·K·mol^−1^ at 1.8 K. Finally, for the Yb analogue [Yb(L)(ox)(H_2_O)], the χ*T* product decreases slowly as the temperature decreases to reach 1.61 emu·K·mol^−1^ at 1.8 K. This behavior is ascribed to the depopulation of the low lying states (*m_J_* states) arising from the ^6^H_5/2_ (Dy^3+^), ^5^I_8_ (Ho^3+^) and ^2^F_7/2_ (Yb^3+^), ground states split through the action of the crystal field (for these ions, the ground state is well below the first excited *J* state). Using the free-ion approach and the isotropic (*z*) component of the susceptibility, the value of the zero-field splitting (ZFS), Δ, was evaluated for each ion using the expressions E2, E3 and E4 in Supporting Information File 1, leading to Δ = 0.169(3), 0.284(4) and 3.25(1) cm^−1^ for Dy, Ho and Yb, respectively. These values are in the range of those reported in the literature [51]. It can be noticed that the introduction of a *zJ*′ term to fit the magnetic curves down to low temperatures for [Dy(L)(ox)(H_2_O)], [Ho(L)(ox)(H_2_O)] and [Yb(L)(ox)(H_2_O)] compounds did not lead to better results.

### Luminescence properties in the solid state

**Figure 4:**
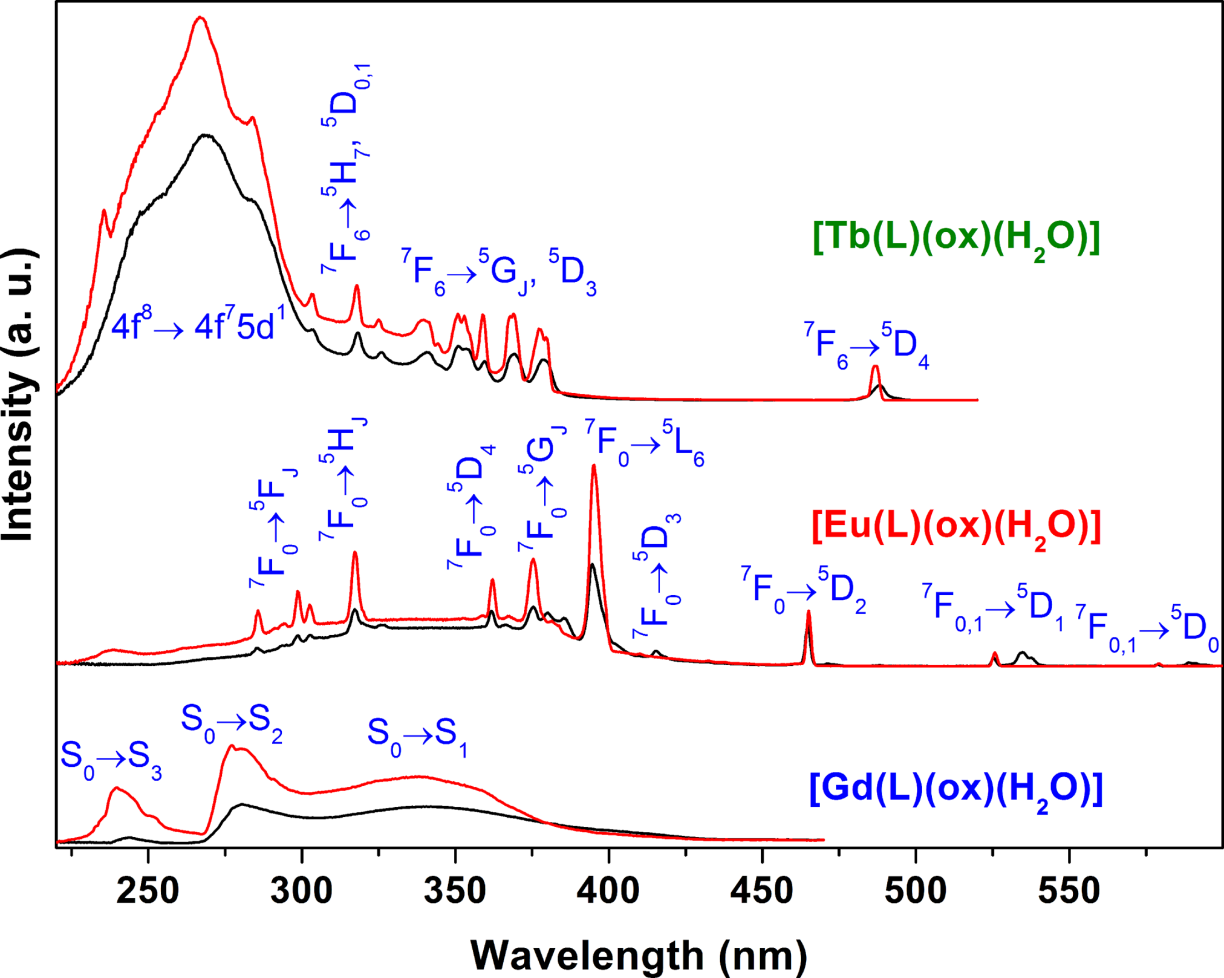
Excitation spectra of [Gd(L)(ox)(H_2_O)] (λ_Em_ = 520 nm), [Eu(L)(ox)(H_2_O)] (λ_Em_ = 619.6 nm) and [Tb(L)(ox)(H_2_O)] (λ_Em_ = 542 nm) recorded at 297 K (black lines) and 12 K (red lines). The intensity is only comparable for the variation of the temperature in each compound.

The excitation spectra of [Gd(L)(ox)(H_2_O)], [Eu(L)(ox)(H_2_O)] and [Tb(L)(ox)(H_2_O)] were recorded at room temperature (ca. 297 K) and 12 K monitoring the ligand emission at 520 nm, and the strongest Eu^3+ 5^D_0_→^7^F_2_ and Tb^3+ 5^D_4_→^7^F_5_ transitions (Figure 4). The [Gd(L)(ox)(H_2_O)] excitation spectra consist of three distinct broad UV bands, ranging from 230 to 400 nm, attributed to the S_0_→S_3,2,1_ excited transitions of the organic ligand. For [Eu(L)(ox)(H_2_O)], these ligand transitions are partially superimposed with the intra-4f^6 7^F_0,1_→^5^D_1-4_, ^5^L_6_, ^5^G_2-6_, ^5^H_3-7_ and ^5^F_1-5_ transitions of Eu^3+^, which dominate the corresponding excitation spectra. Finally, the [Tb(L)(ox)(H_2_O)] excitation spectra feature a strong and broad UV band ranging from 220 to ca. 300 nm, with a maximum at 267 nm, which has no counterpart in the [Gd(L)(ox)(H_2_O)] excitation spectra. Thus, this band is attributed to the inter-configurational spin-forbiden 4f^8^→4f^7^5d^1^ transition of Tb^3+^ because its energy is similar to the energy reported for layered Tb^3+^ silicates [60]. The additional sharp lines in the spectra of [Tb(L)(ox)(H_2_O)] are ascribed to the intra-4f^8 7^F_6_→^5^D_2-4_, ^5^G_J_ and ^5^H_7_ transitions of Tb^3+^. Although with a lower relevance, the excited states of the ligands also contribute to the entire excitation spectra of Tb^3+^, as shown below.

**Figure 5:**
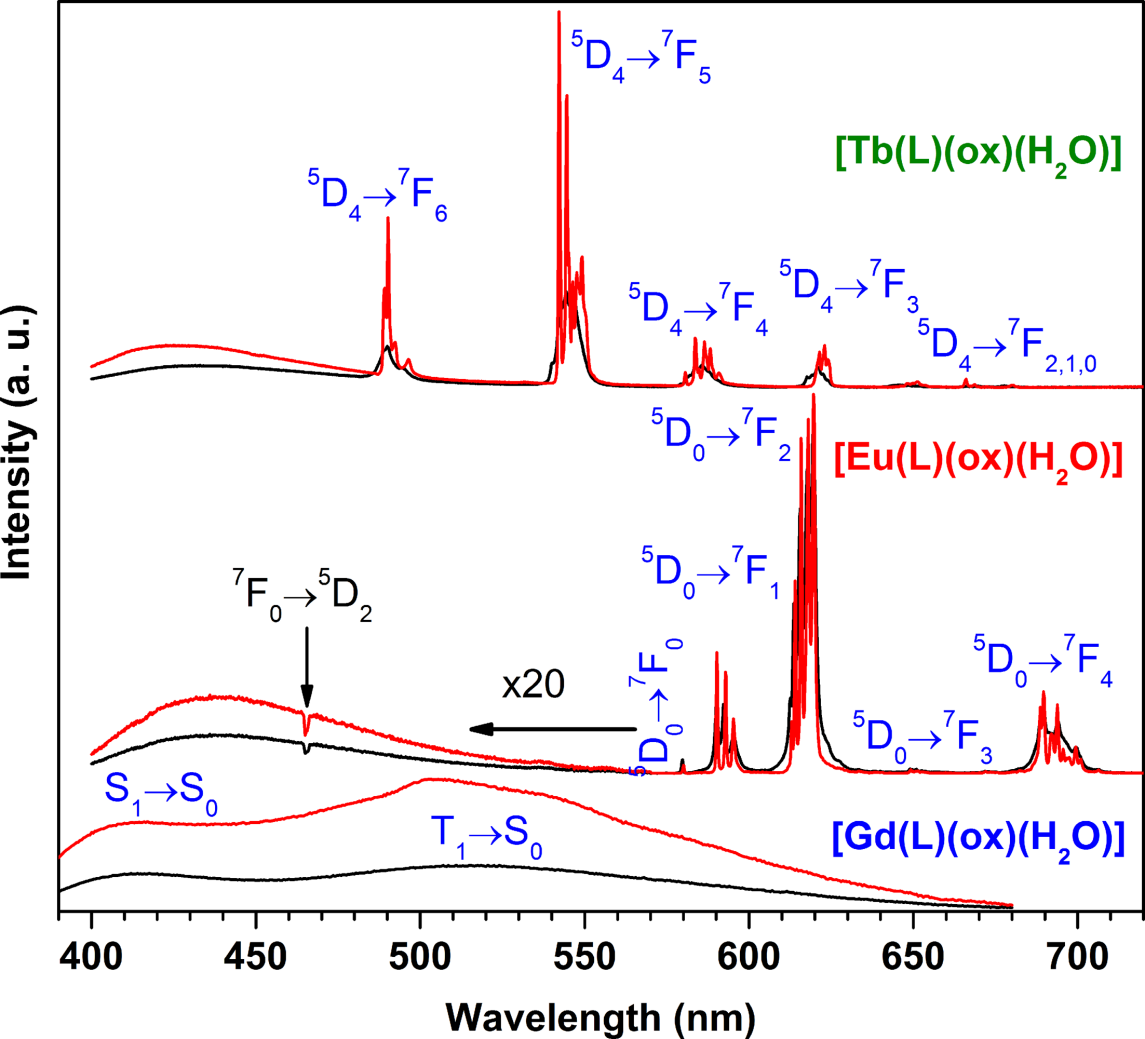
Emission spectra of [Gd(L)(ox)(H_2_O)] (λ_Exc_ = 350 nm), [Eu(L)(ox)(H_2_O)] (λ_Exc_ = 364 nm) and [Tb(L)(ox)(H_2_O)] (λ_Em_ = 364 nm) recorded at 297 K (black lines) and 12 K (red lines). The intensity is only comparable for the variation of the temperature in each compound. For [Eu(L)(ox)(H_2_O)], the negative peak at 465 nm is due to the Eu^3+^ auto-absorption from the ^7^F_0_→^5^D_2_ excited transition.

The emission spectra of [Gd(L)(ox)(H_2_O)], [Eu(L)(ox)(H_2_O)] and [Tb(L)(ox)(H_2_O)] recorded at 297 K and 12 K are given in Figure 5. [Gd(L)(ox)(H_2_O)] displays two broad bands from 390 to ca. 650 nm attributed to the S_1_→S_0_ (peaking at ca. 415 nm) fluorescence and T_1_→S_0_ (peaking at 503 nm) ligand phosphorescence. This assignment is supported by the time-resolved emission spectra recorded at 12 K excited at 350 nm (Figure S6, Supporting Information File 1), which demonstrates a much faster time dependence of the S_1_→S_0_ transition compared to the transition T_1_→S_0_. Under 364 nm excitation, a relative minimum for the Eu^3+^ and Tb^3+^ auto-absorption, [Eu(L)(ox)(H_2_O)] and [Tb(L)(ox)(H_2_O)] show mainly the typical sharp Eu^3+^ and Tb^3+^ emission lines assigned to the ^5^D_0_→^7^F_0-4_ and ^5^D_4_→^7^F_6-0_ transitions, respectively. In addition, both compounds also exhibit a broad band from 400 to ca. 550 nm, particularly weak in the case of the former, attributed to the S_1_→S_0_ transition of the ligand. Accordingly, as exemplified in Figure S7 (Supporting Information File 1) with the [Tb(L)(ox)(H_2_O)] time-resolved 12 K emission spectra, the broad band has a very fast time dependence totally suppressed by a time delay of only 0.05 ms. The suppression of the low-energy T_1_→S_0_ ligand emission denotes an energy transfer from the triplet excited state to the Eu^3+^ and Tb^3+^ excited levels. This energy transfer is more effective for the Eu^3+^ compound, which almost suppresses also the S_1_→S_0_ emission. Under excitation at their corresponding maxima, 270 and 395 nm for Tb^3+^ and Eu^3+^, respectively, both [Eu(L)(ox)(H_2_O)] and [Tb(L)(ox)(H_2_O)] show only the respective sharp emission lines (Figure S8; Supporting Information File 1).

The emission of Eu^3+^ is highly sensitive to slight changes in the first coordination sphere of the metal, and because of this it is widely used as a local probe [61]. For [Eu(L)(ox)(H_2_O)], the emission spectra recorded at 297 K and 12 K show i) a single ^5^D_0_→^7^F_0_ transition and a local-field splitting of the ^7^F_1,2_ levels into three and five Stark components, respectively; ii) and the predominance of the ^5^D_0_→^7^F_2_ transition relatively to the ^5^D_0_→^7^F_1_ one, witnessing the presence of a single low-symmetry Eu^3+^ environment, in accordance with the crystal structure. Additionally, the room-temperature Eu^3+ 5^D_0_ and Tb^3+ 5^D_4_ decay curves were well fitted by single exponential functions, yielding lifetimes of 0.60 ± 0.01 and 0.98 ± 0.01 ms for [Eu(L)(ox)(H_2_O)] and [Tb(L)(ox)(H_2_O)], respectively (Figure S9, Supporting Information File 1), corroborating the presence of a unique Ln^3+^ crystallographic site.

Consideration of these luminescence results prompted the study of isostructural mixed lanthanide networks. In particular, our interest was focused on networks bearing Tb^3+^ and Eu^3+^ in view of their potential application in optical thermometry [17,62]. Accordingly, four Tb^3+^/Eu^3+^ mixed lanthanide networks of formula [Tb_1−_*_x_*Eu*_x_*(L)(ox)(H_2_O)] with *x* = 0.01, 0.03, 0.05 and 0.10 have been synthesized using the same protocol but varying the molar ratio of Tb(NO_3_)_3_·6H_2_O and Eu(NO_3_)_3_·6H_2_O. As expected, these mixed lanthanide networks are isostructural with the parent compound [Ln(L)(ox)(H_2_O)] (Figure S10, Supporting Information File 1) and show the presence of Tb and Eu in the expected ratio and homogeneously distributed in the crystals (Figure S11, Supporting Information File 1).

Consider the emission spectra of the four Tb^3+^/Eu^3+^ mixed lanthanide networks measured at room temperature (Figure S12, Supporting Information File 1). To maximize the relative poor ligand emission, 364 nm excitation was used since it corresponds to a maximum of the ligand excitation and to relative minima of both Eu^3+^ and Tb^3+^ auto-absorptions, as demonstrated by the selective 12 K excitation spectra of [Tb_0.90_Eu_0.10_(L)(ox)(H_2_O)] (Figure S13, Supporting Information File 1). The room-temperature emission spectra of [Tb_0.90_Eu_0.10_(L)(ox)(H_2_O)] under ambient pressure and after exposure to high vacuum (5 × 10^−3^ mbar, Figure S14, Supporting Information File 1) demonstrates the good stability of the emission of the sample against UV irradiation and pressure change.

**Figure 6:**
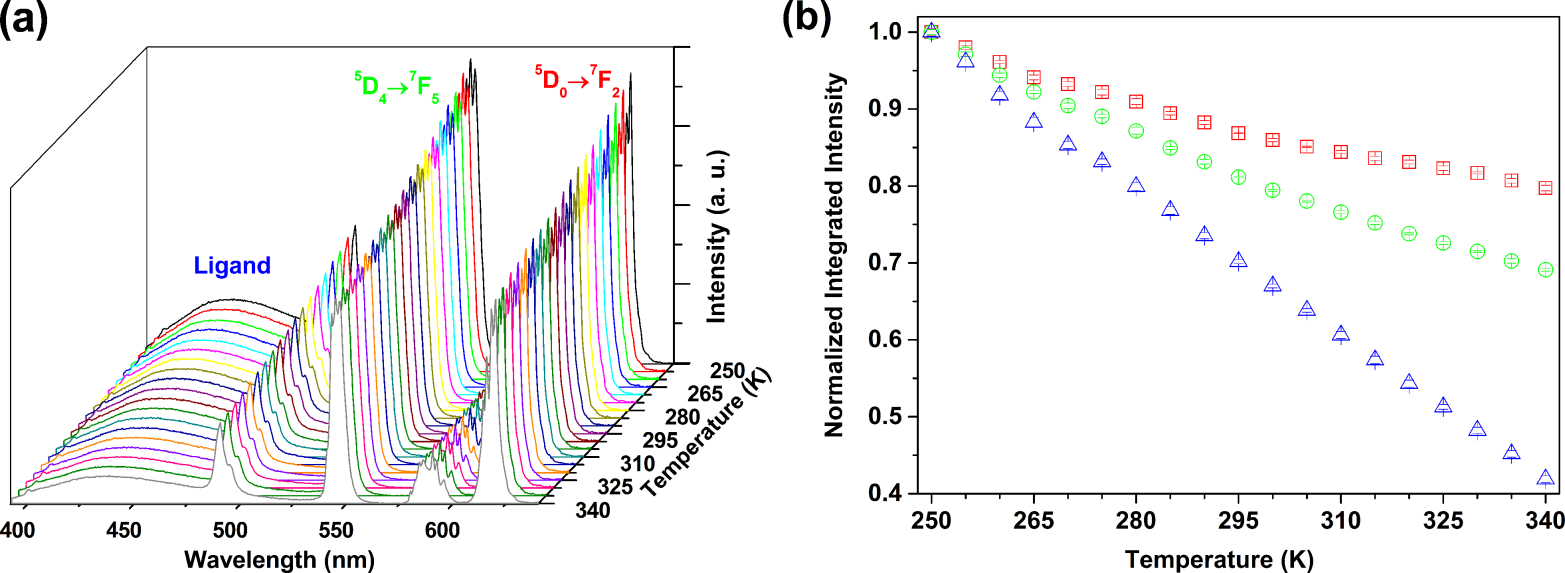
(a) Emission spectra of [Tb_0.97_Eu_0.03_(L)(ox)(H_2_O)] in the range of 250–340 K with the excitation fixed at 364 nm, and (b) corresponding temperature dependence of *I*_L_ (blue), *I*_Tb_ (green) and *I*_Eu_ (red).

Among the four mixed Tb^3+^/Eu^3+^ mixed lanthanide networks, [Tb_0.97_Eu_0.03_(L)(ox)(H_2_O)] presents at room temperature the best balance between the emissions of ligand, Tb^3+^ and Eu^3+^. Based on the integrated areas of the ligand (*I*_L_), Tb^3+ 5^D_4_→^7^F_5_ (*I*_Tb_) and Eu^3+ 5^D_0_→^7^F_2_ (*I*_Eu_) emissions, three distinct thermometric parameters may be defined, Δ_1_ = *I*_Tb_/*I*_Eu_, Δ_2_ = *I*_Tb_/*I*_L_ and Δ_3_ = *I*_Eu_/*I*_L_, allowing for the conversion of the emission intensities into absolute temperature values. The temperature dependence of the [Tb_0.97_Eu_0.03_(L)(ox)(H_2_O)] emission in the range of 250–340 K is presented in Figure 6a. Four consecutive emission spectra were collected for each temperature and used to determine the average thermometric parameter, with the errors calculated from the corresponding standard deviation (95% confidence). *I*_L_, *I*_Tb_ and *I*_Eu_ were determined by integrating the emission spectra in the ranges of 392–478 nm, 536–556 and 606–630 nm, respectively. Figure 6b depicts the temperature dependence of the three integrated emissions. The emission of the ligand decreases by 58% from 250 to 340 K, the Tb^3+^ and Eu^3+^ emissions decrease by 31% and by 20%, respectively.

**Figure 7:**
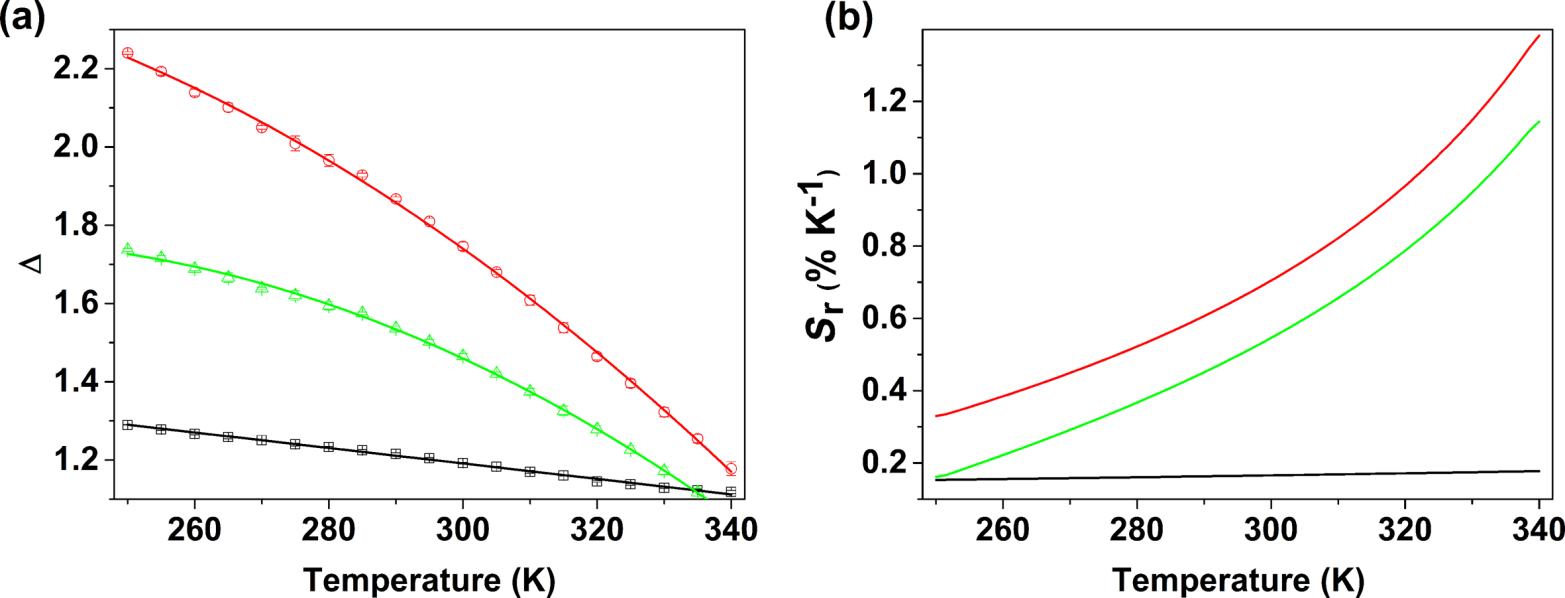
(a) Temperature dependence of Δ_1_ (black), Δ_2_ (green) and Δ_3_ (red) in the range of 250–340 K for [Tb_0.97_Eu_0.03_(L)(ox)(H_2_O)]. The solid lines are the calibration curves, resulting from the fit considering a linear function for Δ_1_, Δ(*T*) = Δ_0_ + *mT* (*r*^2^ = 0.998) and second-order polynomial functions, Δ(*T*) = Δ_0_ + a_1_*T* + a_2_*T^2^*, for Δ_2_ (*r*^2^ = 0.999) and Δ_3_ (r^2^ = 0.999). The bars depict the errors in the thermometric parameter resulting from the propagation of the errors determined for *I*_L_, *I*_Tb_ and *I*_Eu_ and (b) corresponding relative thermal sensitivities in the same temperature range.

The temperature dependence of the thermometric parameters Δ_1_, Δ_2_ and Δ_3_ in the range of 250–340 K is shown in Figure 7a. The corresponding relative sensitivity, defined as *S*_r_ = |∂Δ/∂*T*|/Δ [63], a figure of merit used to compare the performance of ratiometric luminescent thermometers, is plotted in Figure 7b. Δ_2_ and Δ_3_ exhibit very good sensitivities in the range of 250–340 K with maximum relative sensitivities, *S*_m_, of 1.14% and 1.38%·K^−1^ at 340 K, respectively. For Δ_1_, *S*_m_ is only 0.18%·K^−1^. The *S*_r_ values obtained for Δ_3_, in particular, are among the highest reported for metal-organic frameworks or MOF-based luminescent thermometers operative in the physiological range. Indeed, to the best of our knowledge, only eight such visible luminescent ratiometric LnMOF thermometers have been reported [64–71], among which two outperform our material [Tb_0.97_Eu_0.03_(L)(ox)(H_2_O)]: Tb_0.995_Eu_0.005_@In(OH)(2,2′-bipyridine-5,5′-dicarboxylate) with *S*_m_ = 4.47%·K^−1^ at 333 K [65] and Eu@UiO-(2,2′-bipyridine-5,5′-dicarboxylate) with *S*_m_ = 2.19%·K^−1^ at 293 K [67] (value recalculated and corrected using the published calibration curve). Two other thermometers have a performance similar to ours, Eu_0.089_Tb_0.9911_[2,6-di(2′,4′-dicarboxylphenyl)pyridine] with *S*_m_ = 1.39%·K^−1^ at 328 K [70] and [(Eu_0.231_Tb_0.769_(adipate)_0.5_(phthalate)(H_2_O)_2_] with *S*_m_ = 1.21%·K^−1^ at 303 K [71]. These systems are, thus, appealing for potential application as biological sensors [63,72]. The Tb^3+^-to-Eu^3+^ energy transfer plays an important role in the higher sensitivity of Δ_3_ (*I*_Eu_/*I*_L_). On the one hand, the Tb^3+^ lifetimes obtained for [Tb_0.97_Eu_0.03_(L)(ox)(H_2_O)] from single exponential functions (Figure S15, Supporting Information File 1) decrease from 0.98 ± 0.01 ms obtained for the Tb^3+^-only sample at 297 K to 0.62 ± 0.01 ms (250 K) and 0.57 ± 0.01 ms (340 K). On the other hand, the Eu^3+^ lifetimes for the mixed compound (Figure S15, Supporting Information File 1), 0.86 ± 0.02 ms (250 K) and 0.78 ± 0.01 ms (340 K), increases relatively to the one obtained at 297 K for the Eu^3+^-only compound (0.60 ± 0.01 ms). In addition, the Eu^3+^ decay curves also exhibit a rise, to 0.81 ± 0.08 ms (250 K) and 0.93±0.06 ms (340 K), most probably originating from the population of the Eu^3+ 5^D_0_ emitting level trough the Tb^3+ 5^D_4_ donor level.

## Conclusion

Six novel coordination networks based on an imidazolium dicarboxylate 1,3-(biscarboxymethyl)imidazolium and Ln^3+^ ions (Ln = Eu^3+^, Gd^3+^, Tb^3+^, Dy^3+^, Ho^3+^ and Yb^3+^) in the presence of oxalate have been obtained by solvothermal reaction and totally characterized. These coordination networks are isostructural and present a monoclinic structure (space group *P*2_1_/*a*). They exhibit magnetic and luminescent properties that are characteristic for the considered lanthanide ions (except for compounds based on Gd^3+^ ions). The possibility to obtain Tb^3+^/Eu^3+^ mixed lanthanide networks has been exploited for potential application in thermometry. Accordingly, four mixed lanthanide networks [Tb_1−_*_x_*Eu*_x_*(L)(ox)(H_2_O)] (*x* = 0.01, 0.03, 0.05 and 0.10) were synthesized with different Tb^3+^/Eu^3+^ ratios. Using as the thermometric parameter the ratio between the Eu^3+^, ^5^D_0_→^7^F_2_ transition, and the ligand emissions, [Tb_0.97_Eu_0.03_(L)_2_(ox)(H_2_O)] was found to be one of the best three luminescent ratiometric LnMOF thermometers, operative in the physiological range with a maximum sensitivity of 1.38%·K^−1^ at 340 K. The fact that the structure and properties of these coordination networks can be predicted by design constitutes a promising approach to new multifunctional materials, especially magnetic and luminescent, materials.

## Experimental

### Synthesis

Glycine, paraformaldehyde, oxalic acid, Nd(NO_3_)_3_·6H_2_O, Sm(NO_3_)_3_·6H_2_O, Eu(NO_3_)_3_·6H_2_O, Gd(NO_3_)_3_·6H_2_O, Tb(NO_3_)_3_·6H_2_O, Dy(NO_3_)_3_·5H_2_O, Ho(NO_3_)_3_·5H_2_O and Yb(NO_3_)_3_·*x*H_2_O were purchased from Alfa Aesar and were used as received.

[HL] was synthesized according protocols published in the literature [41–42]. Synthesis method and characterizations (elemental analysis, ^1^H and ^13^C NMR) can be found in a previously published paper [31].

[Ln(L)(ox)(H_2_O)] compounds with Ln = Eu^3+^, Gd^3+^, Tb^3+^, Dy^3+^, Ho^3+^ and Yb^3+^ were prepared by solvothermal reaction by mixing [HL] (0.5 mmol), lanthanide nitrate (0.5 mmol) and oxalic acid (0.25 mmol) in a water/ethanol solution (1.5 mL). The solution was sealed in a Teflon-line stainless steel bomb (6 mL) and heated at 393 K for 72 h. After cooling to room temperature, the bomb was opened and colorless crystals were filtered and washed with ethanol and dried at room temperature. Yields were between 41% and 59%. Elemental analysis confirmed the composition of each compounds. [Eu(L)(ox)(H_2_O)]: Anal. calcd for C_9_H_9_N_2_O_9_Eu (440.96 g·mol^−1^): C, 24.49; H, 2.04; N, 6.35; found: C, 24.01; H, 2.06; N, 6.00; [Gd(L)(ox)(H_2_O)]: anal. calcd for C_9_H_9_N_2_O_9_Gd (446.25 g·mol^−1^): C, 24.20; H, 2.02; N, 6.27; found: C, 23.96; H, 2.05; N, 6.24; [Tb(L)(ox)(H_2_O)]: anal. calcd for C_9_H_9_N_2_O_9_Tb (447.92 g·mol^−1^): C, 24.11; H, 2.01; N, 6.25; found: C, 23.68; H, 2.07; N, 6.10; [Dy(L)(ox)(H_2_O)]: anal. calcd for C_9_H_9_N_2_O_9_Dy (451.50 g·mol^−1^): C, 23.92; H, 1.99; N, 6.20; found: C, 23.47; H, 2.05; N, 6.15; [Ho(L)(ox)(H_2_O)]: anal. calcd for C_9_H_9_N_2_O_9_Ho (453.93 g·mol^−1^): C, 23.79; H, 1.98; N, 6.17; found: C, 23.16; H, 2.03; N, 6.05; [Yb(L)(ox)(H_2_O)]: anal. calcd for C_9_H_9_N_2_O_9_Yb (462.04 g·mol^−1^): C, 23.37; H, 1.95; N, 6.06; found: C, 23.08; H, 2.10; N 5.95.

[Tb_1−_*_x_*Eu*_x_*(L)(ox)(H_2_O)] compounds with *x* = 0.01, 0.03, 0.05 and 0.10 were prepared in a similar manner but terbium nitrate and europium nitrate were introduced with the adapted stoichiometry. Yields were between 32% and 34%.

### Physical measurements

Elemental analyses for C, H, N were carried out at the Service de Microanalyses of the Institut de Chimie de Strasbourg. The SEM images were obtained with a JEOL 6700F (scanning electron microscope (SEM) equipped with a field-emission gun (FEG), operating at 3 kV in the SEI mode instrument. FTIR spectra were collected on a Perkin Elmer Spectrum Two UATR-FTIR spectrometer. TGA-TDA experiments were performed using a TA instrument SDT Q600 (heating rates of 5 °C·min^−1^ under air stream). NMR spectra in solution were recorded using a Bruker AVANCE 300 (300 MHz) spectrometer. The emission and excitation spectra were recorded on a modular double grating excitation spectrofluorometer with a TRIAX 320 emission monochromator (Fluorolog-3, Horiba Scientific) coupled to a R928 or a H9170 Hamamatsu photomultiplier, for the detection on the visible and near-infrared spectral ranges, respectively, using the front-face acquisition mode. The excitation source was a 450 W Xe arc lamp. The emission spectra were corrected for detection and optical spectral response of the spectrofluorometer and the excitation spectra were corrected for the spectral distribution of the lamp intensity using a photodiode reference detector. Time-resolved measurements were carried out with the pulsed Xe–Hg lamp excitation, in front-face acquisition mode. The temperature was controlled with a helium closed-cycle cryostat with vacuum system (ca. 5 × 10^−6^ mbar) and a Lakeshore 330 auto-tuning temperature controller with a resistive heater. The temperature can be adjusted from ca. 12 to 450 K with a maximum accuracy of 0.1 K. The sample temperature was fixed to a particular value using the auto-tuning temperature controller; after waiting 5 min to thermalize the sample, four consecutive steady-state emission spectra were measured for each temperature; the maximum temperature difference detected during the acquisitions was 0.1 K, the temperature accuracy of the controller. Magnetic measurements were performed using a Quantum Design SQUID-VSM magnetometer. The static susceptibility measurements were performed in the temperature range of 1.8–300 K with an applied field of 0.5 T. Samples were blocked in eicosane to avoid orientation under magnetic field. Magnetization measurements at different fields and at given temperature confirm the absence of ferromagnetic impurities. Data were corrected for the sample holder and eicosane and diamagnetism was estimated from Pascal constants. The powder XRD patterns were collected with a Bruker D8 diffractometer (Cu Kα_1_, λ = 1.540598 Å) operating at 40 kV and 40 mA equipped with a LynxEye detector. The X-ray diffraction data on single crystal were collected with graphite-monochromatized Mo Kα radiation (λ = 0.71073 Å) with a Kappa Nonius CCD diffractometer at room temperature. Intensity data were corrected for Lorenz-polarization and absorption factors. The structures were solved by direct methods using SIR92 [73], and refined against *F*^2^ by full-matrix least-squares methods using SHELXL-2014 with anisotropic displacement parameters for all non-hydrogen atoms [74–75]. All calculations were performed by using the crystal structure crystallographic software package WINGX [76]. The structure was drawn using Mercury or Diamond [77–78]. Hydrogen atoms were located on a difference Fourier map and introduced into the calculations as a riding model with isotropic thermal parameters. Crystallographic data for the structures reported have been deposited in the Cambridge Crystallographic Data Centre with CCDC reference numbers 1541843, 1541844, 1541845, 1541846, 1541847, 1541848 for [Gd(L)(ox)(H_2_O)], [Yb(L)(ox)(H_2_O)], [Dy(L)(ox)(H_2_O)], [Ho(L)(ox)(H_2_O)], [Tb(L)(ox)(H_2_O)], [Eu(L)(ox)(H_2_O)].

## Supporting Information

Supporting Information contains a representation of the coordination polyhedron, a table of selected bonds, a comparison of the experimental powder X-ray diffraction patterns of the different compounds and the simulated pattern from single crystals X-ray data, SEM analysis, TGA/TDA analysis, a summary of the weight loss values for the different compounds, infrared spectra, luminescence measurement and magnetic expression.

File 1 Additional experimental data.
