# Supplementary material for: Magnetic and luminescent coordination networks based on imidazolium salts and lanthanides for sensitive ratiometric thermometry
Source: Beilstein J Nanotechnol. 2018 Oct 30;9:2775–87. doi: 10.3762/bjnano.9.259 (PMC6244178; doi:10.3762/bjnano.9.259)
Supplement: File 1 — Additional experimental data. [file Beilstein_J_Nanotechnol-09-2775-s001.pdf]

Supporting Information

for

Magnetic and luminescent coordination networks based on imidazolium salts and lanthanides for sensitive ratiometric thermometry

Pierre Farger[1,#], Cédric Leuvrey[1], Mathieu Gallart[1], Pierre Gilliot[1], Guillaume Rogez[1], João Rocha[2], Duarte Ananias[2], Pierre Rabu*[1] and Emilie Delahaye*[1]

Address: [1]Institut de Physique et Chimie des Matériaux de Strasbourg, Université de Strasbourg, CNRS UMR 7504, F-67034 Strasbourg Cedex 2, France; [2]Department of Chemistry, CICECO, University of Aveiro, 3810-193 Aveiro, Portugal and [#]Current address: Laboratoire de Physique et Chimie des Nano-Objets, Institut National des Sciences Appliquées, 135 avenue de Rangueil, 31077 Toulouse Cedex 4, France

Email: Emilie Delahaye* - emilie.delahaye@ipcms.unistra.fr;

Pierre Rabu* - pierre.rabu@ipcms.unistra.fr

* Corresponding author

Additional experimental data

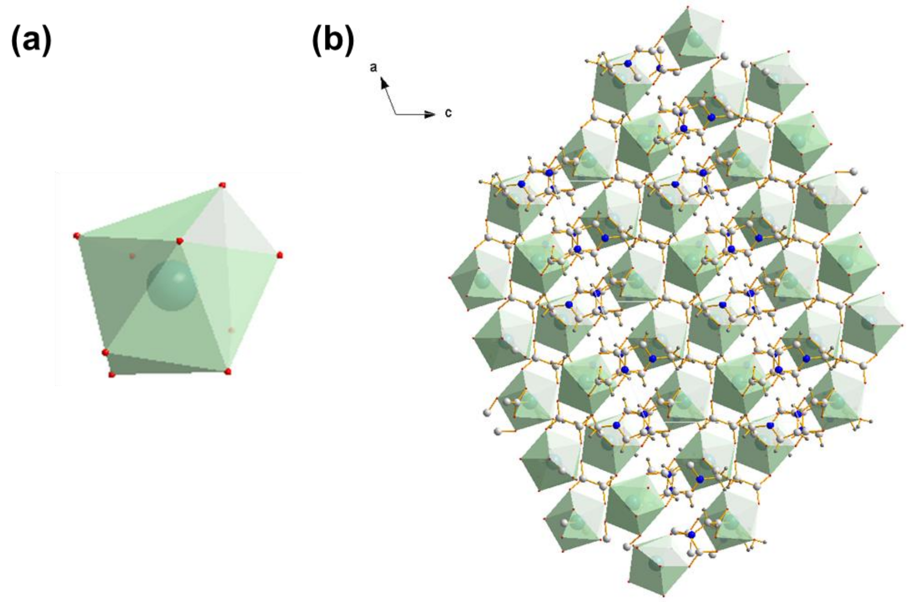

Figure S1: (a) Coordination polyhedron around the Gd^{3+} ion and (b) packing of these coordination polyhedra in the compound [Gd(L)(ox)(H_2O)].

Table S1: Selected bond lengths in Å for the [Ln(L)(ox)(H_2O)] compounds with Ln = Eu^{3+}, Gd^{3+}, Tb^{3+}, Dy^{3+}, Ho^{3+} and Yb^{3+}.

	Eu^{3+}	Gd^{3+}	Tb^{3+}	Dy^{3+}	Ho^{3+}	Yb^{3+}
Ln1–O1	2.514(4)	2.542(3)	2.547(5)	2.459(4)	2.437(6)	2.405(8)
Ln1–O2	2.555(4)	2.486(3)	2.473(5)	2.522(4)	2.523(5)	2.494(7)
Ln1–O3	2.489(4)	2.504(3)	2.541(5)	2.477(4)	2.468(6)	2.413(8)
Ln1–O4	2.552(4)	2.551(3)	2.492(5)	2.528(4)	2.520(6)	2.334(7)
Ln1–O5	2.401(4)	2.419(3)	2.377(4)	2.390(4)	2.385(5)	2.314(7)
Ln1–O6	2.425(4)	2.384(3)	2.402(4)	2.351(4)	2.347(5)	2.354(8)
Ln1–O7	2.389(3)	2.392(3)	2.411(5)	2.394(4)	2.380(6)	2.299(8)
Ln1–O8	2.422(4)	2.415(3)	2.367(4)	2.358(4)	2.386(6)	2.332(8)
Ln1–O9	2.428(4)	2.396(3)	2.390(5)	2.366(4)	2.345(6)	2.519(9)
Ln–$O_{average}$	2.463	2.454	2.444	2.428	2.421	2.385
Ln–$Ln_{shortest}$	6.21	6.19	6.17	6.13	6.12	6.04

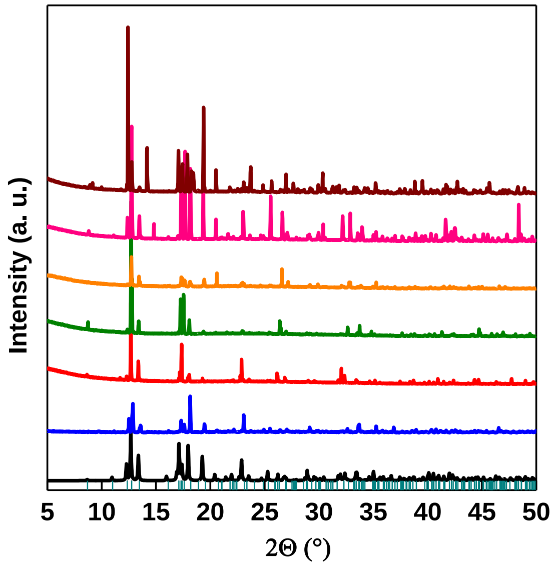

Figure S2: Comparison of the experimental powder X-ray diffraction patterns for the compounds [Gd(L)(ox)(H_2O)] (blue line), [Eu(L)(ox)(H_2O)] (red line), [Tb(L)(ox)(H_2O)] (green line), [Dy(L)(ox)(H_2O)] (orange line), [Ho(L)(ox)(H_2O)] (pink line), [Yb(L)(ox)(H_2O)] (brown line) and the simulated pattern from single crystals X-ray data of the compound [Tb(L)(ox)(H_2O)] (black line). The green vertical lines indicate the position of the calculated diffraction lines.

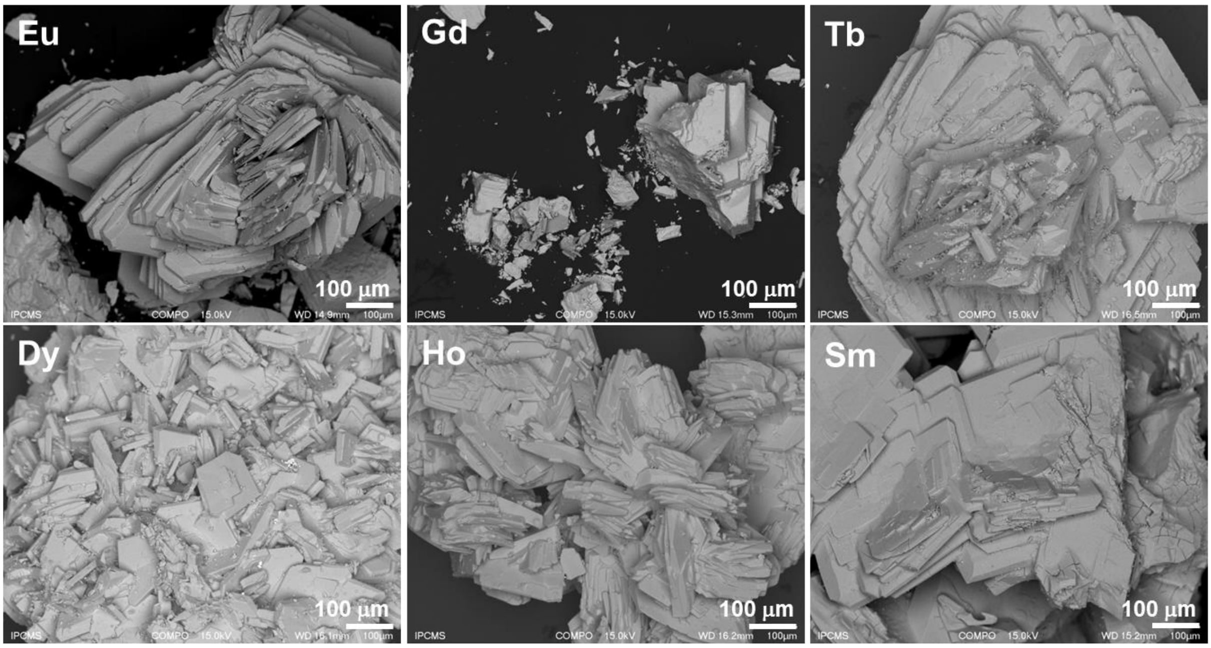

Figure S3: SEM images in composition of the [Ln(L)(ox)(H_2O)] compounds with Ln = Eu^{3+}, Gd^{3+}, Tb^{3+}, Dy^{3+}, Ho^{3+} and Yb^{3+}.

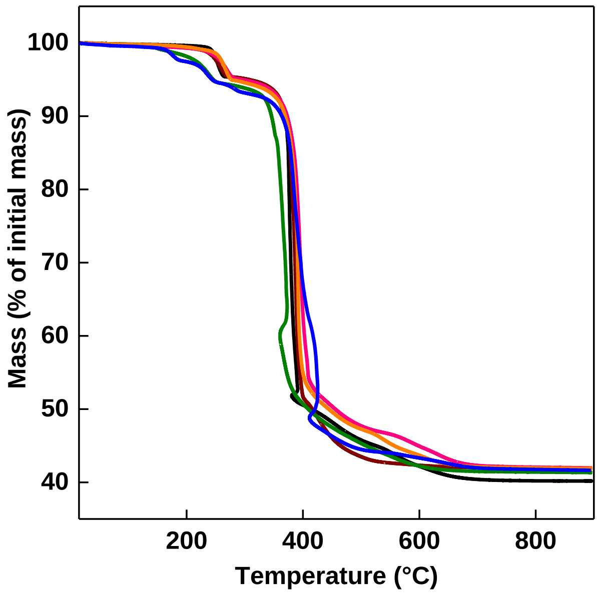

Figure S4: TGA curves of [Tb(L)(ox)(H_2O)] (blue line), [Ho(L)(ox)(H_2O)] (pink line), [Eu(L)(ox)(H_2O)] (green line), [Dy(L)(ox)(H_2O)] (red line), [Yb(L)(ox)(H_2O)] (orange line), [Gd(L)(ox)(H_2O)] (black line).

Table S2: Summary of the weight loss values for the [Ln(L)(ox)(H_2O)] compounds with Ln = Eu^{3+}, Gd^{3+}, Tb^{3+}, Dy^{3+}, Ho^{3+} and Yb^{3+}.

	**Eu**	**Gd**	**Tb**	**Dy**	**Ho**	**Yb**
**step 1 : Elimination of water molecule**						
observed	5.80%	4.85%	4.80%	5.25%	5.05%	5.40%
calculated	4.08%	4.03%	4.02%	3.99%	3.97%	3.90%
**step 2 : combustion of organic moieties concomitant with formation of oxide**						
observed	56.10%	57.75%	56.03%	55.78%	55.79%	55.96%
calculated	58.40%	57.67%	57.45%	56.98%	56.66%	55.62%
**final oxide**	Eu_2O_3	Gd_2O_3	Tb_2O_3	Dy_2O_3	Ho_2O_3	Yb_2O_3

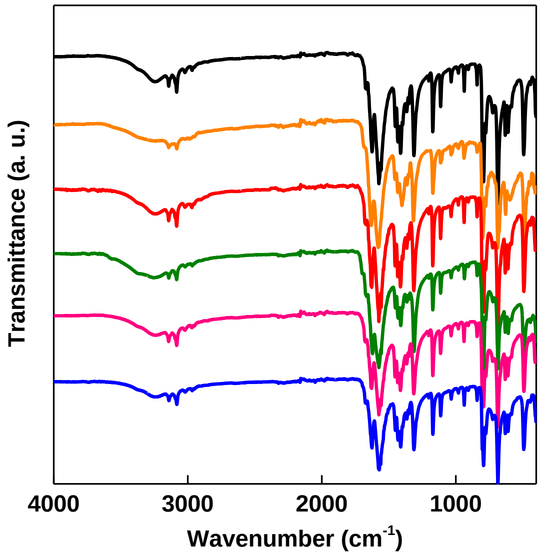

Figure S5: Infrared spectra of [Tb(L)(ox)(H_2O)] (blue line), [Ho(L)(ox)(H_2O)] (pink line), [Eu(L)(ox)(H_2O)] (green line), [Dy(L)(ox)(H_2O)] (red line), [Yb(L)(ox)(H_2O)] (orange line), [Gd(L)(ox)(H_2O)] (black line).

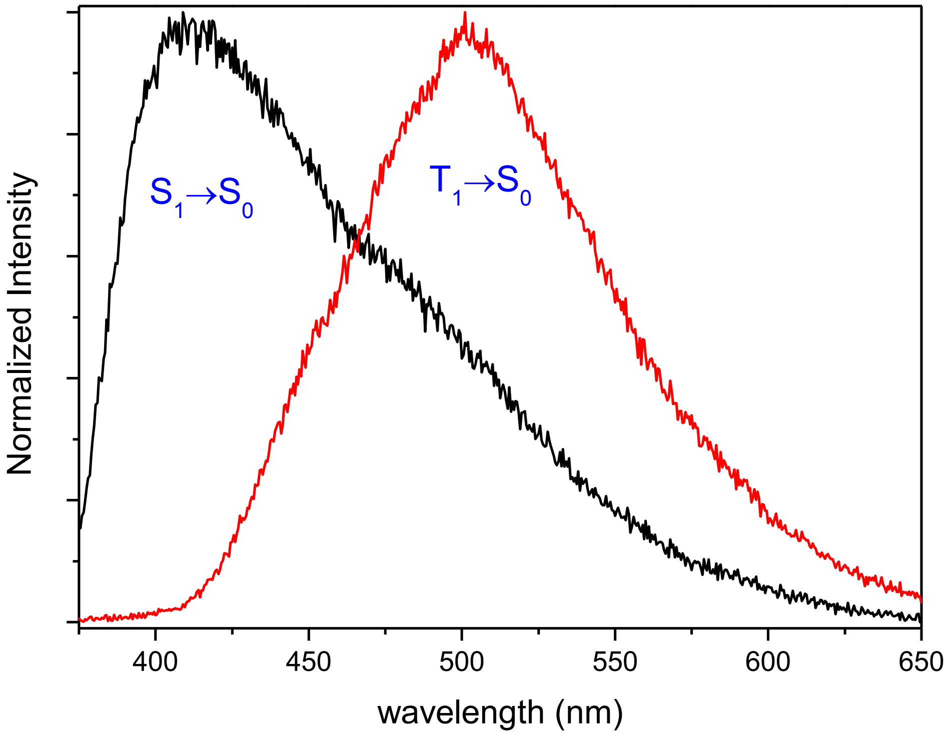

Figure S6: Time-resolved emission spectra of [Gd(L)(ox)(H_2O)] measured at 12 K with 350 nm excitation. Black line: initial delay of 0.01 ms and integration time of 0.2 ms; red line: initial delay of 0.2 ms and integration time of 10 ms. Time-resolved emission spectra were not corrected for detection and optical spectral response of the spectrofluorometer.

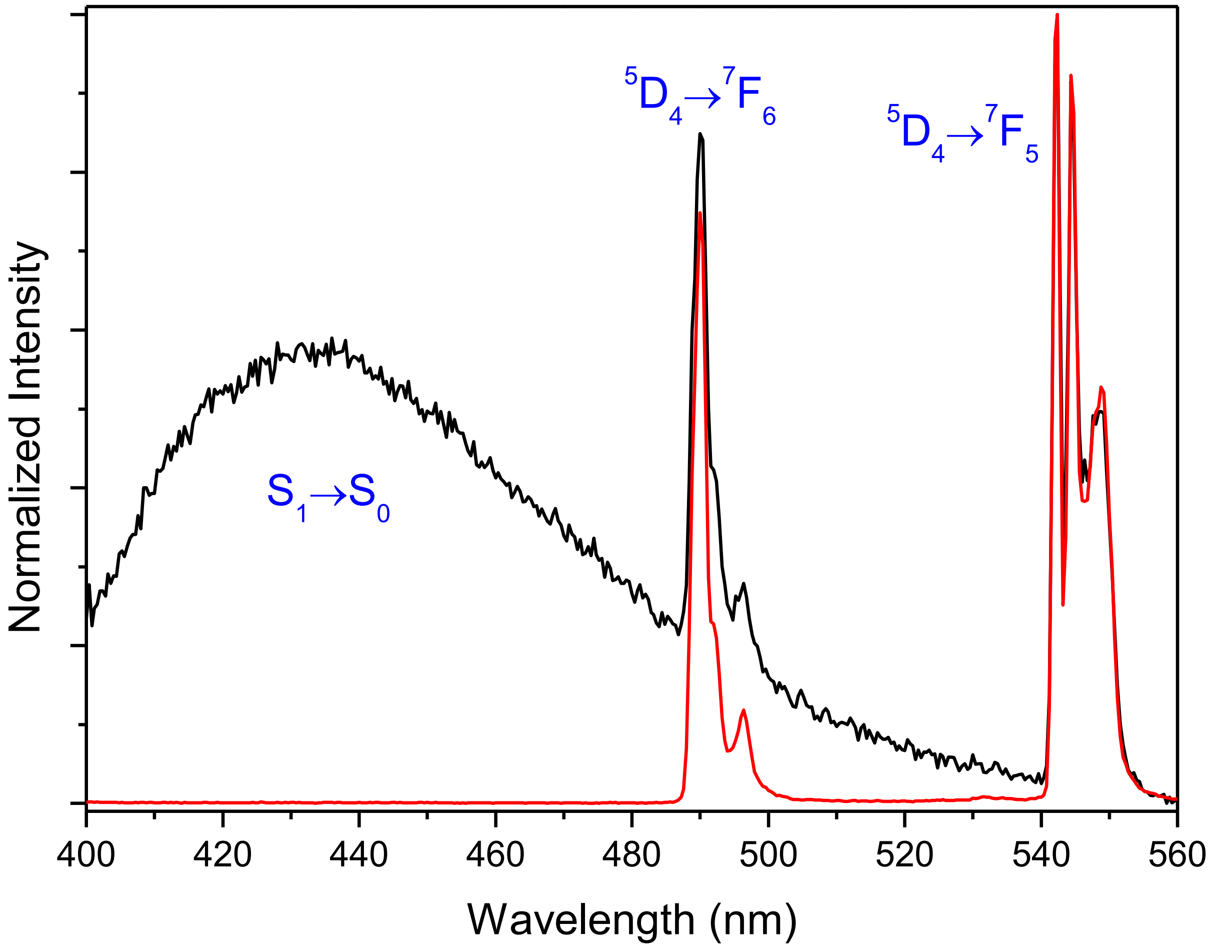

Figure S7: Selected region of the12 K time-resolved emission spectra of [Tb(L)(ox)(H_2O)] measured with 364 nm excitation. Black line: initial delay of 0.01 ms and integration time of 0.1 ms; red line: initial delay of 0.05 ms and integration time of 5 ms. Time-resolved emission spectra were not corrected for detection and optical spectral response of the spectrofluorometer.

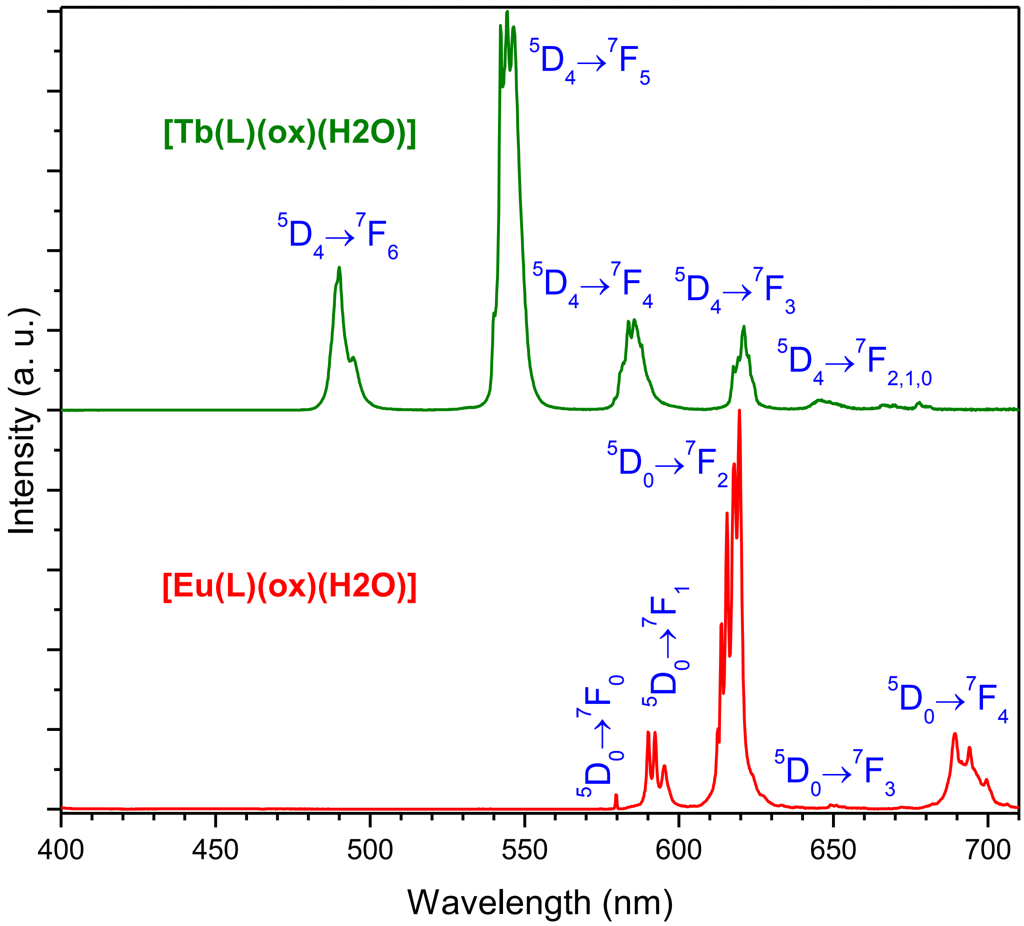

Figure S8: Emission spectra of [Eu(L)(ox)(H_2O)] (red line; λ_{Exc} = 395 nm) and [Tb(L)(ox)(H_2O)] (green line; λ_{Exc} = 270 nm) recorded at 297 K.

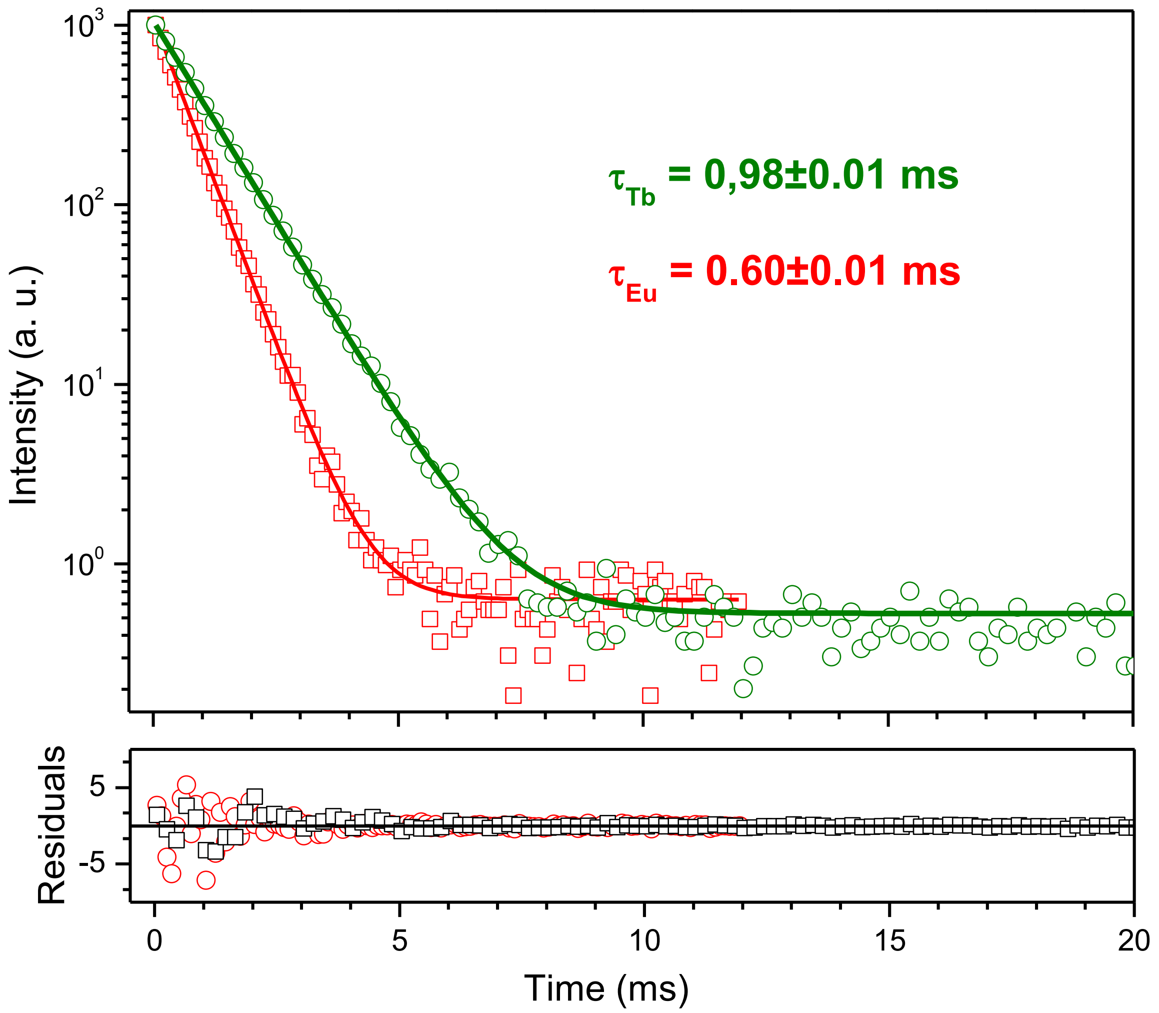

Figure S9: $^5D_0$ and $^5D_4$ decay curves of [Eu(L)(ox)(H_2O)] (λ_{Exc} = 395 nm, red) and [Eu(L)(ox)(H_2O)] ($\lambda_{Exc.}$ = 488 nm, green), measured at 297 K, monitoring the Eu^{3+} and Tb^{3+} emission at 619.6 nm and 542 nm, respectively, and fitted with single exponential decay functions ($r^2 > 0.999$).

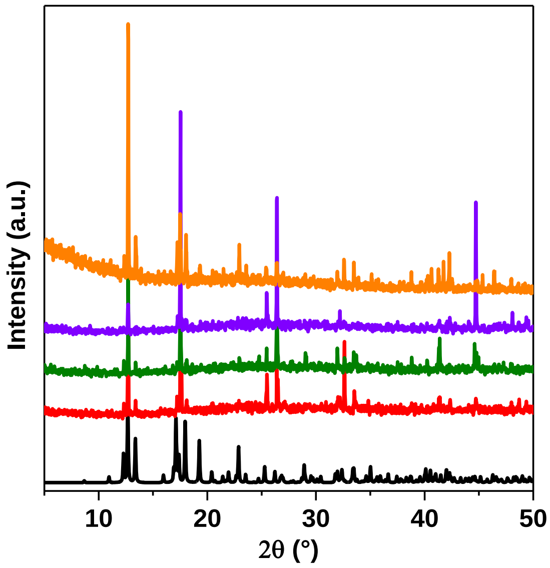

Figure S10: Comparison of the experimental powder X-ray diffraction patterns for the compounds [$Tb_{1-x}Eu_x(L)(ox)(H_2O)$] with x = 0.01 (red line), 0.03 (green line), 0.05 (violet line) and 0.10 (orange line), and the simulated pattern from single crystals X-ray data of the compound [$Tb(L)(ox)(H_2O)$] (black line).

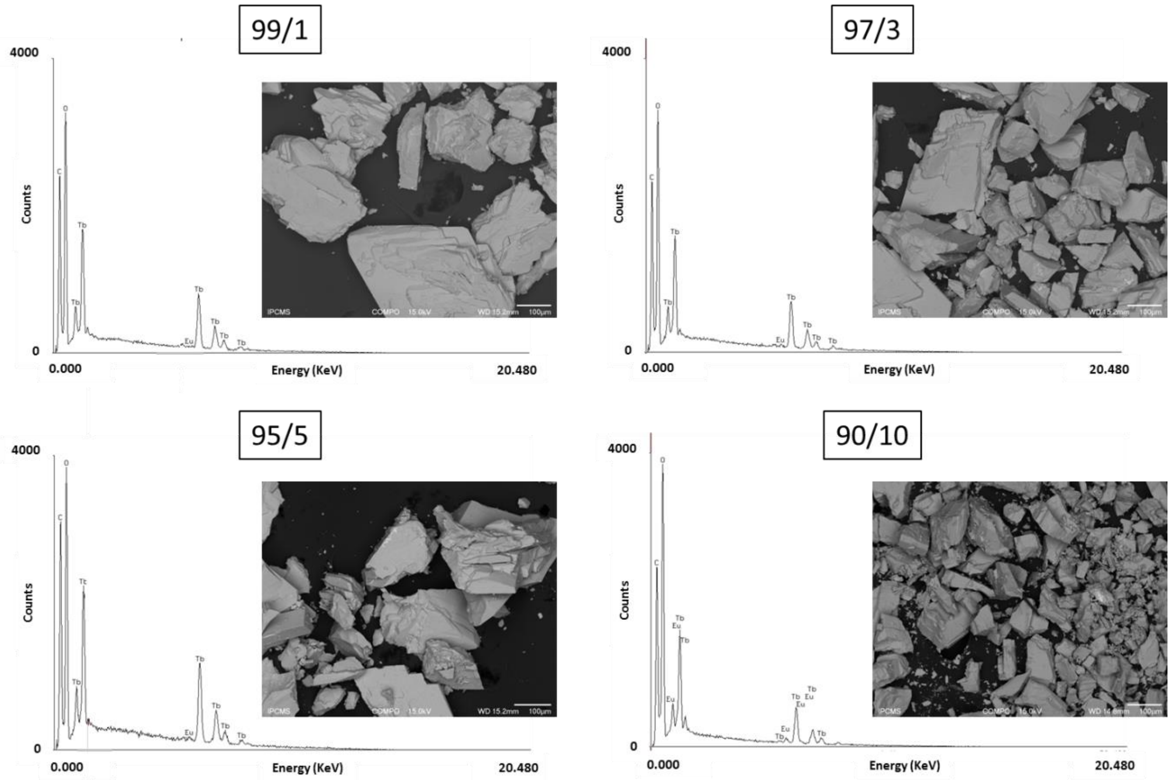

Figure S11: SEM images in composition and EDX spectra of the compounds [$Tb_{1-x}Eu_x(L)_2(ox)(H_2O)$] with x = 0.01, 0.03, 0.05 and 0.10.

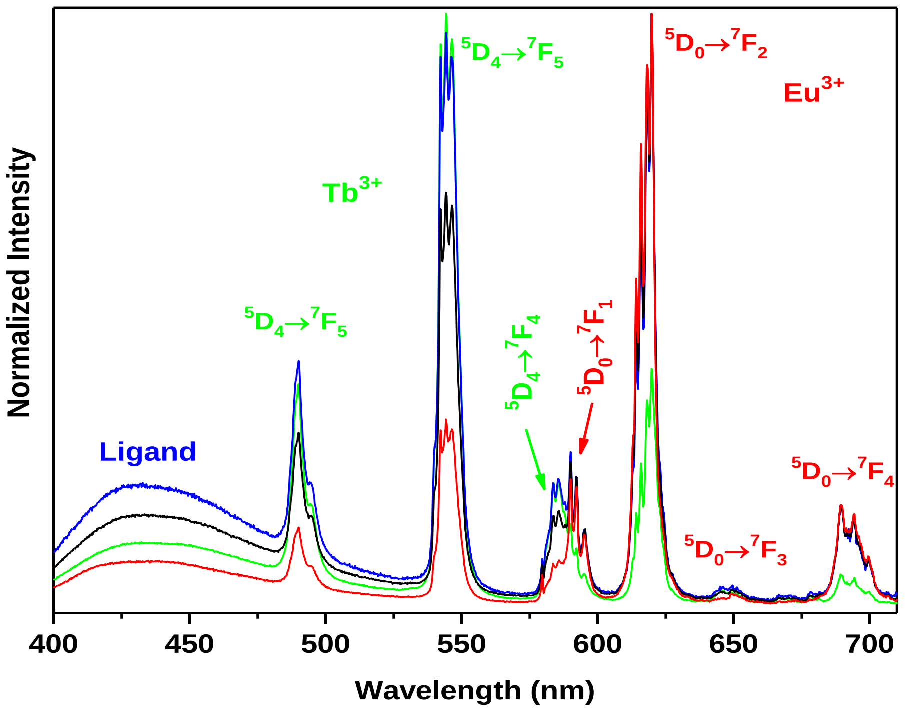

Figure S12: Room-temperature emission spectra of [$Tb_{1-x}Eu_x(L)_2(ox)(H_2O)$] with the excitation fixed at 364 nm, for x = 0.01 (green line), 0.03 (blue line), 0.05 (black line) and 0.10 (red line).

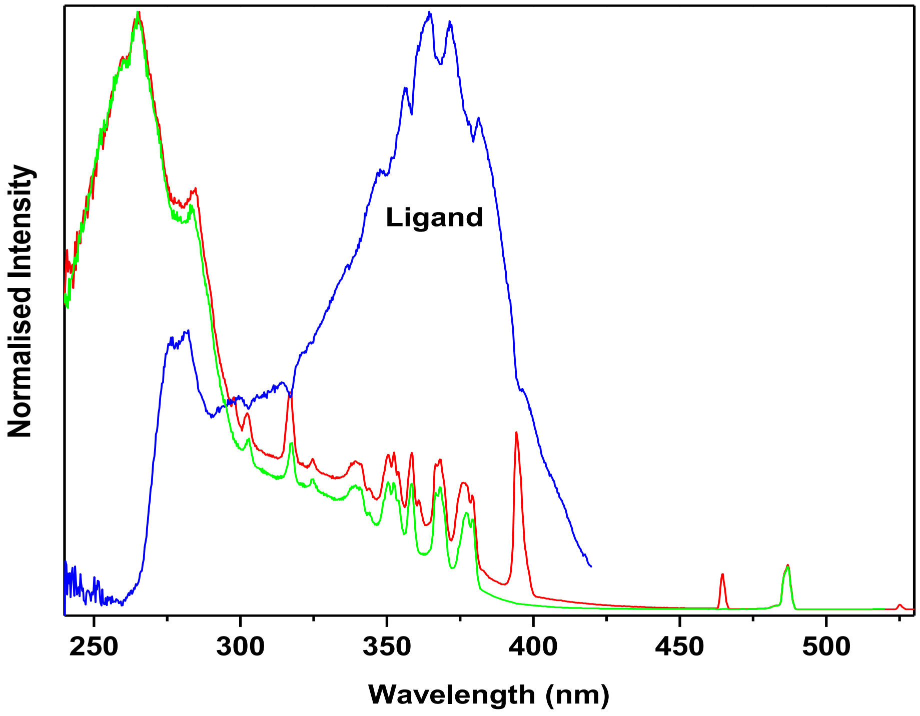

Figure S13: Excitation spectra of [$Tb_{0.90}Eu_{0.10}(L)_2(ox)(H_2O)$] recorded at 12 K detecting the emissions of Eu^{3+} at 620 nm (red line), Tb^{3+} at 542 nm (green line) and of the ligand at 450 nm (blue line).

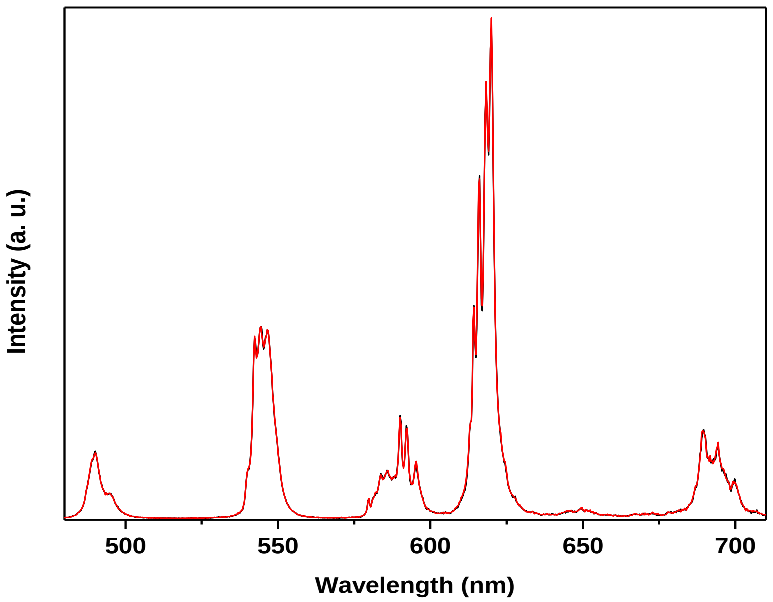

Figure S14: Room-temperature emission spectra of [$Tb_{0.90}Eu_{0.10}(L)_2(ox)(H_2O)$] under ambient pressure (1 bar; black line) and in high vacuum (5×10^{-3} mbar; red line) measured with 285 nm excitation.

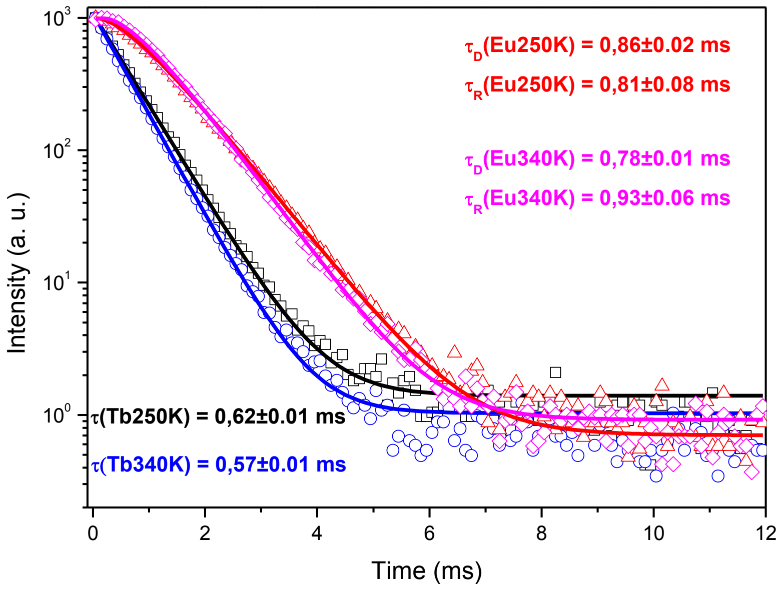

Figure S15: 250 K and 340 K lifetime decay curves of [$Tb_{0.90}Eu_{0.10}(L)_2(ox)(H_2O)$] ($\lambda_{Exc}$ = 364 nm) measured monitoring the Eu^{3+} and Tb^{3+} emission at 619.6 nm and 542 nm, respectively. Tb^{3+} $^5D_4$ decay curves were fitted with single exponential decay functions (r^2 > 0.999), whereas the Eu^{3+} decay curves were fitted with the equation $I = \left[I_0 + I_1\left(1 - \exp\left(-t/\tau_r\right)\right)\right]\exp\left(-t/\tau_d\right) + I_c$, where I and I_0 are the intensities of the emitting level at time t and t = 0, τ_r, is the rise-time of the emitting lower-level, τ_d is the decay time of the emitting level and I_c is the background constant.

Expressions used for the analysis of the magnetic data:

E1

$$\chi = \frac{N\beta^2}{3kTx} * \frac{24+(13.5x-1.5)\exp(-x)+(67.5x-2.5)\exp(-3x)+(189x-3.5)\exp(-6x)+(405x-4.5)\exp(-10x)+(742.5x-5.5)\exp(-15x)+(1228.5x-6.5)\exp(-21x)}{1+3\exp(-x)+5\exp(-3x)+7\exp(-6x)+9\exp(-10x)+11\exp(-15x)+13\exp(-21x)}$$

with N is the Avogadro number, β the Bohr magneton, k the Boltzmann constant and λ the coupling constant and $x = \frac{\lambda}{kT}$.

E2

$$\chi = \frac{Ng^2\beta^2}{kT} * \frac{0.5\exp\left(\frac{-0.25\Delta}{kT}\right)+4.5\exp\left(\frac{-2.25\Delta}{kT}\right)+12.5\exp\left(\frac{-6.25\Delta}{kT}\right)+24.5\exp\left(\frac{-12.25\Delta}{kT}\right)+40.5\exp\left(\frac{-20.25\Delta}{kT}\right)+60.5\exp\left(\frac{-30.25\Delta}{kT}\right)+84.5\exp\left(\frac{-42.25\Delta}{kT}\right)+112.5\exp\left(\frac{-56.25\Delta}{kT}\right)}{1+2\exp\left(\frac{-0.25\Delta}{kT}\right)+2\exp\left(\frac{-2.25\Delta}{kT}\right)+2\exp\left(\frac{-6.25\Delta}{kT}\right)+2\exp\left(\frac{-12.25\Delta}{kT}\right)+2\exp\left(\frac{-20.25\Delta}{kT}\right)+2\exp\left(\frac{-30.25\Delta}{kT}\right)+2\exp\left(\frac{-42.25\Delta}{kT}\right)+2\exp\left(\frac{-56.25\Delta}{kT}\right)}$$

E3

$$\chi = \frac{Ng^2\beta^2}{kT} * \frac{2\exp\left(\frac{-\Delta}{kT}\right)+8\exp\left(\frac{-4\Delta}{kT}\right)+18\exp\left(\frac{-9\Delta}{kT}\right)+32\exp\left(\frac{-16\Delta}{kT}\right)+50\exp\left(\frac{-25\Delta}{kT}\right)+72\exp\left(\frac{-36\Delta}{kT}\right)+98\exp\left(\frac{-49\Delta}{kT}\right)+128\exp\left(\frac{-64\Delta}{kT}\right)}{1+2\exp\left(\frac{-\Delta}{kT}\right)+2\exp\left(\frac{-4\Delta}{kT}\right)+2\exp\left(\frac{-9\Delta}{kT}\right)+2\exp\left(\frac{-16\Delta}{kT}\right)+2\exp\left(\frac{-25\Delta}{kT}\right)+2\exp\left(\frac{-36\Delta}{kT}\right)+2\exp\left(\frac{-49\Delta}{kT}\right)+2\exp\left(\frac{-64\Delta}{kT}\right)}$$

E4

$$\chi = \frac{Ng^2\beta^2}{kT} * \frac{0.5\exp\left(\frac{-0.25\Delta}{kT}\right)+4.5\exp\left(\frac{-2.25\Delta}{kT}\right)+12.5\exp\left(\frac{-6.25\Delta}{kT}\right)+24.5\exp\left(\frac{-12.25\Delta}{kT}\right)}{2\exp\left(\frac{-0.25\Delta}{kT}\right)+2\exp\left(\frac{-2.25\Delta}{kT}\right)+2\exp\left(\frac{-6.25\Delta}{kT}\right)+2\exp\left(\frac{-12.25\Delta}{kT}\right)}$$

E5

$$\chi\mathrm{m} = \frac{N\beta^2}{3kTx} * \frac{1228.5x-6.5+(742.5x-5.5)\exp(6x)+(405x-4.5)\exp(11x)+(189x-3.5)\exp(15x)+(67.5x-2.5)\exp(18x)+(1.53x-1.5)\exp(20x)+6\exp(21x)}{13+11\exp(6x)+9\exp(11x)+7\exp(15x)+5\exp(18x)+3\exp(20x)+\exp(21x)}$$
